# The transcription factor WRKY22 is required during cryo-stress acclimation in Arabidopsis shoot tips

**DOI:** 10.1093/jxb/eraa224

**Published:** 2020-07-25

**Authors:** Johanna Stock, Andrea Bräutigam, Michael Melzer, Gerd Patrick Bienert, Boyke Bunk, Manuela Nagel, Jörg Overmann, E R Joachim Keller, Hans-Peter Mock

**Affiliations:** 1 Leibniz Institute of Plant Genetics and Crop Plant Research (IPK) Gatersleben, Seeland, Germany; 2 Leibniz Institute DSMZ-German Collection of Microorganisms and Cell Cultures, Braunschweig, Germany; 3 Microbiology, Braunschweig University of Technology, Braunschweig, Germany; 4 Royal Holloway, University of London, UK

**Keywords:** Abiotic stress, cryoprotectant, shoot tip, stomatal closure, transcription factor, transcriptomics, ultra-low temperature

## Abstract

Storage of meristematic tissue at ultra-low temperatures offers a mean to maintain valuable genetic resources from vegetatively reproduced plants. To reveal the biology underlying cryo-stress, shoot tips of the model plant *Arabidopsis thaliana* were subjected to a standard preservation procedure. A transcriptomic approach was taken to describe the subsequent cellular events which occurred. The cryoprotectant treatment induced the changes in the transcript levels of genes associated with RNA processing and primary metabolism. Explants of a mutant lacking a functional copy of the transcription factor WRKY22 were compromised for recovery. A number of putative downstream targets of WRKY22 were identified, some related to phytohormone-mediated defense, to the osmotic stress response, and to development. There were also alterations in the abundance of transcript produced by genes encoding photosynthesis-related proteins. The *wrky22* mutant plants developed an open stomata phenotype in response to their exposure to the cryoprotectant solution. WRKY22 probably regulates a transcriptional network during cryo-stress, linking the explant’s defense and osmotic stress responses to changes in its primary metabolism. A model is proposed linking WRKY53 and WRKY70 downstream of the action of WRKY22.

## Teaser

The transcription factor WRKY22 links the explant’s defense and osmotic stress response during Arabidopsis shoot tip cryo-stress.

## Introduction

**Fig. 1.**
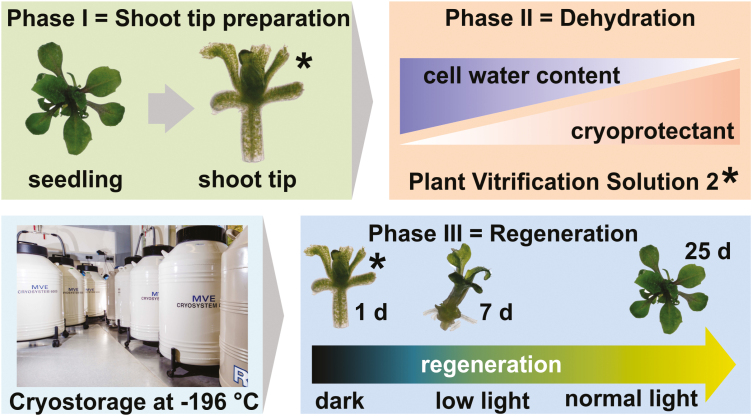
Schematic overview of the long-term storage protocol. Phase I, excision of shoot tips; phase II, gradual reduction in explant hydration by treatment with cryoprotectant; phase III, recovery from cryopreservation over 25 d. PVS2, Plant Vitrification Solution 2. *Sampled for RNA-seq analysis.

Plant biodiversity disappears rapidly as a direct impact of humankind’s use of plant natural resources. It is of tremendous importance to preserve genetic resources, which are otherwise irretrievably lost. In plant gene banks, several *ex situ* plant conservation and propagation techniques, starting from seed storage, in field maintenance, tissue culture, or long-term storage at ultra-low temperatures, so-called cryopreservation, were established (Engelmann and Takagi, 2000). Plant storage at ultra-low temperatures, between –140 °C and –196 °C, is frequently used in the context of maintaining plants which can only be reproduced vegetatively or to preserve selected clonal material/varieties of heterozygous plant species (Panis *et al.*, 1996; Wu *et al.*, 2003; Keller, 2005). The storage procedure imposes a spectrum of abiotic stresses, including wounding following the preparation of the explant from its mother plant, osmotic stress occurring as a result of the dehydration, and the chemical toxicity of the cryoprotectant itself. During cryo-storage, the explant also suffers from a rapid and large variation in temperature. Studies focusing on the regulation of the acclimation to cryo-associated abiotic stress have begun to reveal that oxidative stress is also of relevance (Basu, 2008; El-Banna *et al.*, 2010; Ren *et al*., 2013, 2015; Gross *et al.*, 2017). A range of tissues/organs are used as explants, including non-differentiated callus, cell cultures, buds, and shoot tips (Benson *et al.*, 2007; Reed, 2008). Various protocols were optimized for a number of key gene bank species, seeking to avoid the formation of ice crystals in the tissue, as the expansion associated with freezing can result in severe mechanical damage. This goal is usually supported by treating the explant with cryoprotectants, such as Plant Vitrification Solution 2 (PVS2), before being ultra-rapidly cooled (Fig. 1) (Sakai *et al.*, 1990; Engelmann, 2004).

Currently, there is no rational design of a protocol for cryopreservation, as the molecular framework underlying successful long-term storage of plant meristems is not understood. Shoot tips of the model plant *Arabidopsis thaliana* can be successfully cryopreserved and regenerated, which allows us to take advantage of the extensive genomic and genetic resources developed for this species (Stock *et al*., 2019). Several genes are known to be differentially transcribed during the cryo-stress of *A. thaliana* shoot tips: these include *SQD1* (*At4g33030*), *MBF1C* (*At3g24500*), a gene encoding a putative aspartyl protease (*At1g66180*), *PR5* (*At1g75040*), and the gene encoding the WRKY22 (*At4g01250*) transcription factor (TF) (Gross *et al.*, 2017). However, their functional necessity for cryoprotection was only addressed for WRKY22 in this study.

WRKY22 itself is known to regulate upstream processes of low temperature acclimation (Chawade *et al.*, 2007; Park *et al.*, 2015), hypoxia-induced immunity (Hsu *et al.*, 2013), pathogen-triggered immunity (Dong *et al.*, 2003; Göhre *et al.*, 2012), and leaf senescence (Zhou *et al.*, 2011). Therefore, we hypothesize that WRKY22 has a substantial role when combinations of different abiotic stressors, such as during cryopreservation, appear during plant development.

In our study, we demonstrated that the absence of a functional copy of the WRK22 TF results in a compromised level of post-cryogenic recovery of Arabidopsis and, hence, aimed to model the functional role of WRKY22 when different cryogenic stressors were applied.

The first objective of the present research was to evaluate the molecular events occurring during the cryo-storage of wild-type (WT) *A. thaliana* shoot tips. This was achieved by determining its transcriptome during the preparation stage of the shoot tip explants (phase I), during their cryoprotectant treatment (phase II), and during their rewarming and recovery (phase III) (Fig. 1). The second objective was to characterize the differences between the shoot tip transcriptomes of a *wrky22* knockout mutant (KO) and the WT, with the intention of detecting the downstream transcriptional effects (particularly during phase II) associated with the presence of *WRKY22*. A complex gene regulatory network dependent on the TF WRKY22 was unraveled. Our study demonstrates that Arabidopsis shoot tips are an excellent model to elucidate the molecular crosstalk necessary to cope with cryo-stress.

## Materials and methods

### Plant material and growing conditions

Seeds of the *A. thaliana* ecotype Columbia-0 (WT) and relevant T-DNA insertion lines (see Supplementary Table S1a at *JXB* online) were obtained from The Nottingham Arabidopsis Stock Centre (NASC) (http://arabidopsis.info). The T-DNA insertion lines were validated by a genomic PCR (primer sequences shown in Supplementary Table S1b). Surface-sterilized seeds were plated on solidified Murashige and Skoog (MS) medium (Murashige and Skoog, 1962) containing 3% (w/v) sucrose, and held for 21 d under an 8 h photoperiod (light intensity 150 µmol m^–2^ s^–1^) with a day/night temperature regime of 22/20 °C. After this, the seedlings were shifted to a 22/8 °C regime for 3 weeks to provide the explants used for cryo-storage. The full-length coding sequence of *WRKY22* was amplified from cDNA and cloned into the binary vector pB2GW7.0 (Karimi *et al.*, 2002) under the control of the *Cauliflower mosaic virus* (CaMV) 35S promoter (*35S:comp*) using the Gateway system (www.thermofisher.com), according to the supplier’s protocol. The vector was introduced into the *wrky22.1* mutant using the *Agrobacterium*-mediated floral dip method (Bechtold *et al.*, 1993). Successfully transformed plants were identified using Basta selection, supported by a reverse transcription real-time PCR (RT–PCR) assay directed at *WRKY22*. The relevant primer sequences are given in Supplementary Table S1b.

### Transmission electron microscopy

For comparative ultrastructural analysis, Arabidopsis apical shoot meristem tissue of 2–3 mm length of the WT and the *wrky22.1* mutant was dissected out and used for microwave-assisted sample preparation. Therefore 3–5 explants of preparation stages I, II, and III have been used for aldehyde/osmium tetroxide fixation, substitution in acetone, embedding in Spurr resin, and sample polymerization as described in Supplementary Table S3. Ultrathin sectioning and ultrastructure analysis were performed as described (Daghma *et al.*, 2011).

### Cryo-storage and regeneration

The chosen protocol was previously described in detail by Stock *et al.* (2017). For experiments using whole seedlings instead of shoot tips, seedlings were treated as conducted for shoot tips. In brief, after an overnight immersion of the excised shoot tips in liquid MS medium (pH 5.8) containing 0.1 M sucrose, the material was partially desiccated for 20 min by immersion in MS medium (pH 5.8) containing 2 M glycerol and 0.4 M sucrose. This solution was then replaced by PVS2 [30% (w/v) glycerol, 15% (w/v) ethylene glycol, 15% (w/v) DMSO, 0.4 M sucrose in MS, pH 5.8] for 1 h at 4 °C in the dark. After treatment with liquid nitrogen, shoot tips were rewarmed and placed onto recovery medium. For regeneration, the explants were maintained in the dark for 3 d at 22 °C, then for 4 d under low-light, long-day conditions (16 h photoperiod, irradiance 20–30 µmol m^–2^ s^–1^, 22/20 °C), and finally under a normal light regime (16 h photoperiod, irradiance 150 µmol m^–2^ s^–1^, 22/20 °C) for an additional 18 d. Visual assessments were made after a recovery period of 25 d: explants showing no sign of any development were considered as ‘dead’; those which regenerated incomplete shoot/root/leaf structures or callus were classed as ‘surviving’; and the third category represented those which developed into normal plants (‘recovered’). Only recovered plantlets were included in the statistical analyses, which were based on the Win Fisher test. The values reported here represent the mean of three replicates, each of which comprised a group of 30 shoot tips.

### RNA extraction, cDNA synthesis, and quantitative RT–PCR

RNA extraction from 3–5 shoot tips at each of the three cryo-stress phases as defined in Fig. 1 (at the end of phases I, II, and III), was performed using an RNeasy Plus Micro Kit (Qiagen GmbH, Hilden, Germany). A 0.5 µg aliquot of DNase I-treated RNA was used as the template for synthesis of the first cDNA strand, using Maxima Reverse Transcriptase, primed by oligo(dT)_18_ (Thermo Fisher Scientific, Waltham, MA, USA). The resulting cDNAs were subjected to qRT–PCRs driven by a variety of gene-specific primers (sequences given in Supplementary Table S1b) in reactions based on SsoAdvanced™ Universal SYBR^®^ Green Supermix (BioRad Laboratories, Hercules, CA, USA). The amplifications were run on a LightCycler® 480 Real Time PCR System (Roche Diagnostics GmbH, Mannheim, Germany), with three technical replicates.

For experiments using whole Arabidopsis seedlings, ~30 mg of Arabidopsis plant material was harvested, immediately frozen in liquid nitrogen, and ground by vortexing four times with four steel beads. Total RNA was extracted using NucleoSpin RNA Plant Kits and DNase I treatment according to the manufacturer’s instructions (Macherey-Nagel, Germany). cDNA was synthesized from 1 µg of total RNA using MuLV-Reverse Transcriptase (Fermentas, Germany) in a total volume of 20 µl and diluted to 1:20 with nuclease-free water. qRT–PCR was performed in a 384-well thermocycler (CFX384 Touch™ Real-Time PCR Detection System, Bio-Rad) using the GoTaq qPCR Mastermix (Promega, USA). Seven identically treated biological replicates were analyzed.

The resulting data were analyzed using QBASEPLUS v2.3 software (Biogazelle, Ghent, Belgium),employing *Clath* (*At5g46630*) and *TIP41* (*At4g34270*) (Czechowski *et al.*, 2005) as reference genes (primer sequences are given in Supplementary Table S1b). Primer specificity was assessed by inspection of a melting curve derived after 40 amplification cycles. For the generation of the standard curves, aliquots of all cDNAs per time point were combined into a mixture. This mixture was serially diluted by 2-fold dilutions down to 1:64 with nuclease-free water to generate standard curve templates and to determine PCR efficiencies for each primer pair.

### RNA-seq analysis

For the purpose of the RNA-seq analysis, RNA was extracted using an RNeasy Plant Mini Kit (Qiagen) from a bulk of 100 shoot tips per replicate (three) per sampling point from both WT and *wrky22.1* mutant shoot tips and treated with DNase. For mRNA purification and poly(A) selection, the Illumina TruSeq RNA Sample Preparation v2 Kit (Illumina, San Diego, CA, USA) was used. Library preparation was performed using the ScriptSeq™ v2 RNA-Seq Library Preparation Kit (Epicentre, Madison, WI, USA) following the manufacturer’s protocol. Quality assessment of the libraries was done using the Agilent 2100 Bioanalyzer (Agilent Technologies, Santa Clara, CA, USA). Cluster generation of the prepared libraries was performed using the cBot (Illumina) and TruSeq SR Cluster Kit v3-cBot-HS (Illumina) following the manufacturer’s instructions. The concentration of libraries loaded in the flowcells was 12 pM, followed by sequencing on a HiSeq 2500 instrument with the TruSeq SBS Kit v3-HS (Illumina) for 50 cycles. Image analysis and base calling were performed using the Illumina pipeline v 1.8.

### Accession numbers

The RNA-seq data from this article have been deposited in the European Nucleotide Archive (http://www.ebi.ac.uk/ena) with the accession number PRJEB22967.

### Read mapping and gene expression profiling, GO term enrichment analysis, and MapMan functional annotation

Single end reads of triplicated WT and KO samples were mapped onto representative *A. thaliana* transcripts (TAIR v10; https://www.arabidopsis.org/) with kallisto (-l 190, -s 20) (Bray *et al.*, 2016). Transcript counts and normalized transcripts per million reads (tpm) were combined and analyzed using R software (www.r-project.org). Differentially expressed genes (DEGs) were determined using the Bioconductor packager edgeR (www.bioconductor.org/packages/release/bioc/html/edgeR.html) (Robinson *et al.*, 2010) including multiple hypothesis testing correction (Bonferroni, 1936) to avoid false positives at the possible expense of power. Gene annotations and ontology were retrieved from TAIR v10 and functional annotations from the Mapman repository (http://mapman.gabipd.org/) (see Supplementary Dataset 3 at *JXB* online). Gene Ontology (GO) term enrichment analyses were conducted with the Bioconductor package topGO (www.bioconductor.org/packages/devel/bioc/html/topGO.html) (Alexa and Rahnenfuhrer, 2010) based on Fisher’s exact test (Fisher, 1922).

### Estimation of stomatal density and aperture

A method adapted from Li *et al.* (2013) was used to estimate stomatal density and aperture from leaves detached from 4-week-old WT, *wrky22.1*, and *wrky22.2* mutant plants. Plants of each of the WT and the *wrky22.1* and *wrky22.2* mutants were soil grown under an 8 h photoperiod (light intensity 150 µmol m^–2^ s^–1^) with a day/night temperature regime of 22/20 °C for 4 weeks. Estimates of stomatal closure were based on observations taken from three biological replicates. The leaves were floated in 30 mM KCl, 10 mM MES-KOH (pH 6.1) under 150 µmol m^–2^ s^–1^ light for 2 h at room temperature, then in the same buffer containing either 10 µM abscisic acid (ABA) for 2 h at room temperature, or PVS2 at 4 °C for 1 h in the dark. Stomatal aperture was represented by the ratio between the stomatal width and length, obtained from a sample of 80 stomata. Stomatal density and aperture were recorded with a Keyence Digital Microscope VHX-5000 (KEYENCE GmbH, Neu-Isenburg, Germany).

### Drought stress experiment

A set of 40 plants of each of the WT or the *wrky22.1* or *wrky22.2* mutants was soil grown under an 8 h photoperiod (light intensity 150 µmol m^–2^ s^–1^) with a day/night temperature regime of 22/20 °C for 4 weeks, then transferred into a cabinet delivering an 8 h photoperiod (light intensity 120 µmol m^–2^ s^–1^), a temperature regime of 22/20 °C, and a relative humidity of 40%. Each pot was initially well watered, after which water was withheld for 0–21 d. At each sampling point, the FW of three plants per genotype was obtained and their rosette diameter measured. Soil moisture was determined using a HH2 Moisture Meter (DELTA-T DEVICES, Cambridge, UK).

### Promoter *in silico* analysis

Prediction of the promoter region and putative *cis*-binding elements for target genes of WRKY22 was conducted using http://PlantPAN2.itps.ncku.edu.tw.

## Results

### The inactivation of *WRKY22* compromises the regrowth of cryopreserved explants

**Fig. 2.**
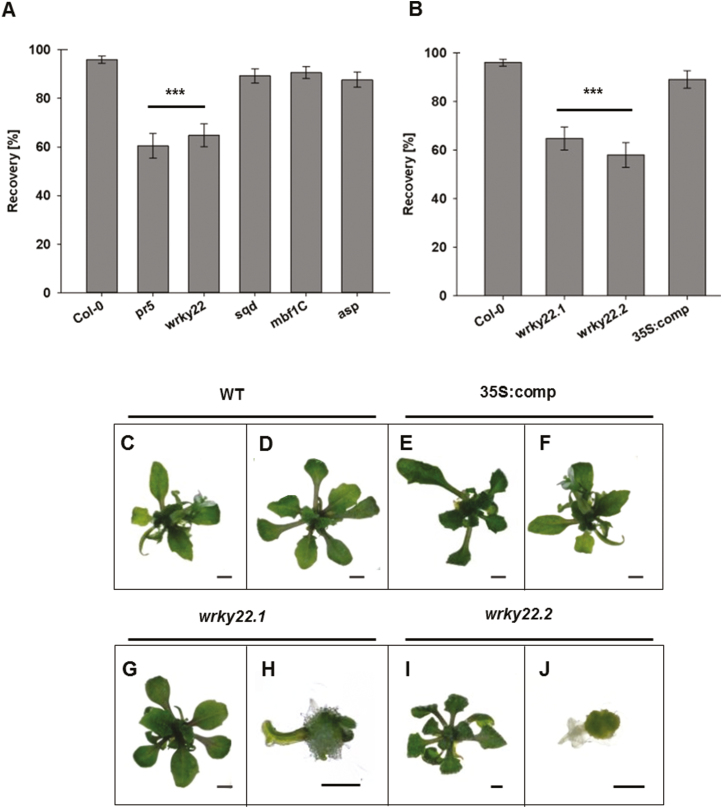
Regrowth of Arabidopsis T-DNA mutant explants. The proportion of recovered plantlets after a 25 d recovery period of (A) the WT and mutants *pr5*, *wrky22*, *sqd1*, *mbf1C*, and *asp*, and (B) the *wrky22.1* and *wrky22.2* mutants and a transgenic *wrky22.1* mutant plant harboring the transgene *35S:comp*. The bars represent the mean % of successful regeneration, with its associated SD. The performance of the mutants was compared with that of the WT using the Win Fisher test (***P≤0.001, *n*≥90). (C–J) The appearance of recovered plantlets derived from shoot tips of (C, D) WT, (E, F) transgenic *wrky22.1* mutant plant harboring the *35S:comp*, (G, H) *wrky22.1* mutant, and (I, J) *wrky22.2* mutant showing (C–G, I) regenerated and (H, J) surviving but non-recovering shoot tips. Scale bar=1 mm.

The effect of knocking out the genes *At4g33030* (*SQD1*), *At3g24500* (*MBF1C*), *At1g66180* (*ASP*), *At1g75040* (*PR5*), and *At4g01250 (WRKY22*) on the performance of cryopreserved explants was explored by exposing T-DNA KO lines for each gene to the cryopreservation protocol. The regeneration capacity of *A. thaliana* shoot tips was analyzed after a cryo-storage of 30 min. As at –196 °C metabolic activity ceases, preservation of the shoot tips will be similar between a short-term (30 min) and a long-term (>2 years) storage. The vast majority (98%) of WT shoot tips regenerated into viable plantlets after cryogenic treatment, as did explants from the mutant lines involving *At4g33030*, *At3g24500*, and *At1g66180*. In contrast, the lack of a fully functional copy of either *WRKY22* or *PR5* resulted in a significantly impaired level of regeneration (Fig. 2A). As little is known about the molecular mechanism relevant for successful cryopreservation, we focused on the role of the TF WRKY22. By testing two independent T-DNA insertion mutants (*wrky22.1* and *wrky22.2*), it was shown that the loss of function of *WRKY22* was responsible for the observed loss in regeneration. In both cases, the genes’ highly conserved WRKY domain sequence was disrupted by a T-DNA sequence, resulting in a highly reduced abundance of *WRKY22* transcripts (Supplementary Fig. S1). The regeneration rate was reduced from 98% for WT explants to 60% for those derived from each of the mutants (Fig. 2B). Introducing a copy of WT *WRKY22* driven by the CaMV 35S promoter (*35S:comp*) into the *wrky22.1* mutant resulted in a *WRKY22* transcript level similar to that measured in the WT explant and restored the WT phenotype (Fig. 2B; Supplementary Fig. S1). Regenerated plantlets from the WT (Fig. 2C, D) and *35S:comp* (Fig. 2E, F) resembled one another with respect to their rosette leaves, roots, and shoots, while the *wrky22* plantlets exhibited a distinct phenotype: 60% of the plantlets retained a WT phenotype (Fig. 2G, I), while 40% developed incomplete leaves and roots, produced some callus material, or stayed green without any further development (Fig. 2H,J).

### PVS2 treatment induces *WRKY22* abundance and ultrastructural changes

**Fig. 3.**
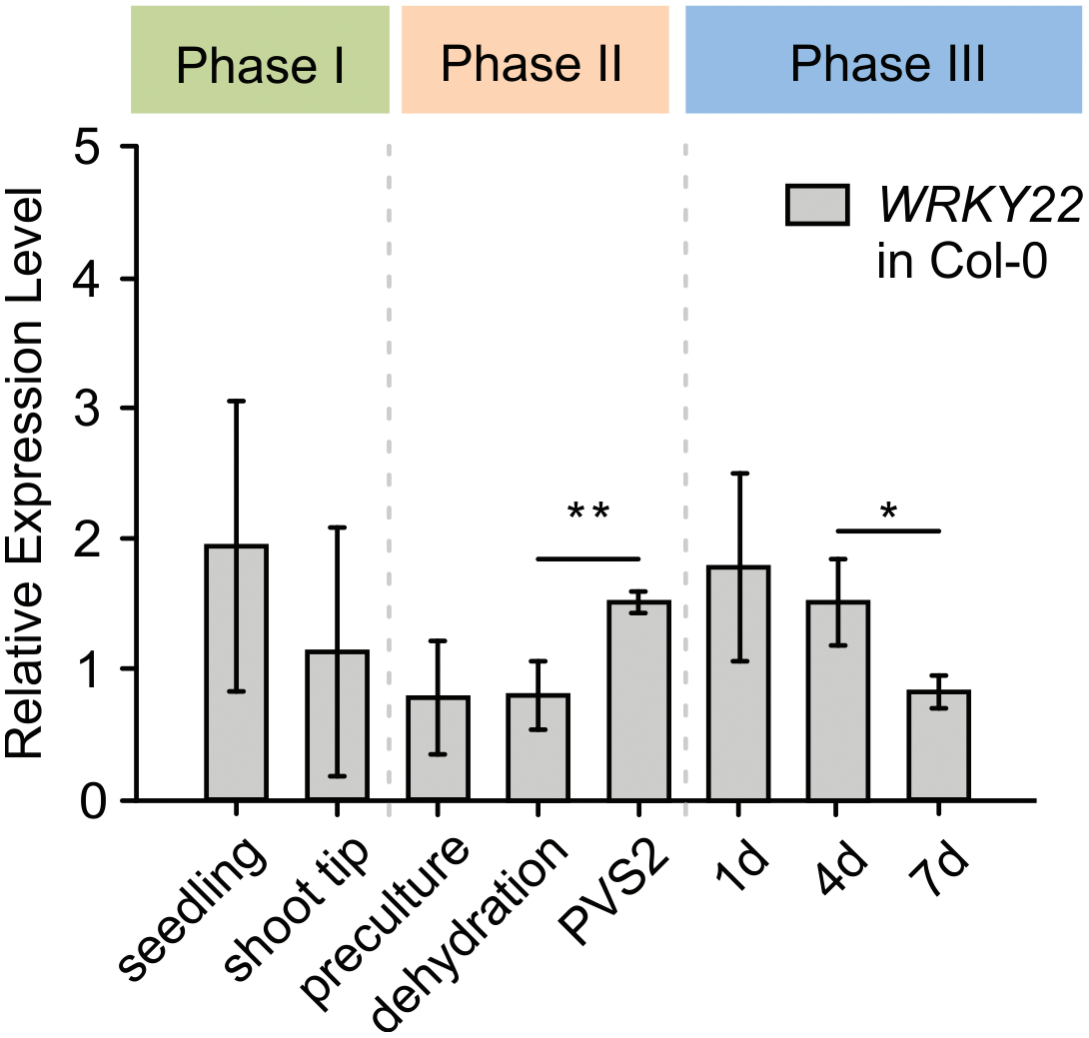
Relative abundance of *WRKY22* transcripts present in Arabidopsis WT explants. Six-week-old Columbia seedlings were sampled at each stages of the cryopreservation/acclimation process. After shoot tip preparation (phase I), stepwise cellular dehydration and cryoprotection using Plant Vitrification Solution 2 (PVS2, phase II), and post-cryogenic recovery (phase III), transcript levels were detected by qRT–PCR using specific primers. The data represent means ±SD from four independent biological replicates (*n*=4). ** and *: means differ at *P*≤0.01 and ≤0.05, respectively, using one-way ANOVA followed by Holm–Sidak post-hoc test.

qRT–PCR of WT explants revealed that *WRKY22* transcription increased significantly over phase II, and decreased after the fourth day of phase III (Fig. 3), indicating that the TF is likely to be involved in the response to PVS2-induced stress and the early phase of regeneration.

**Fig. 4.**
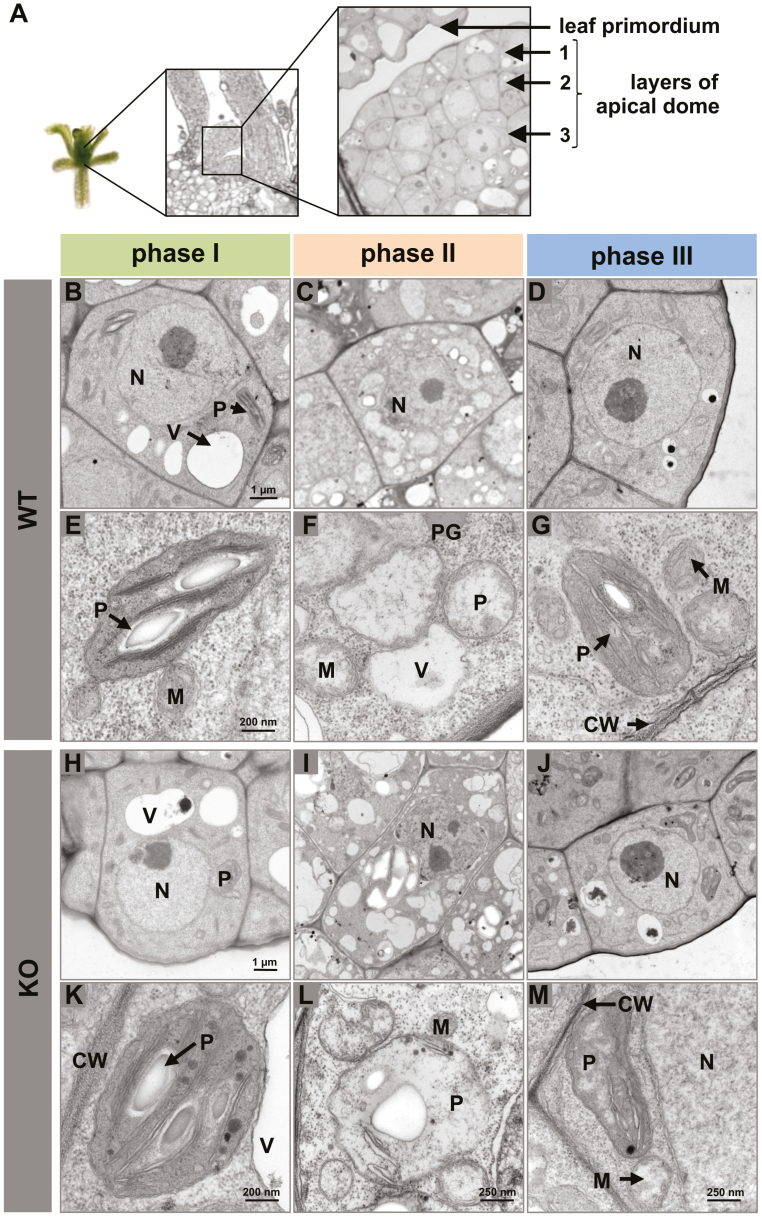
The effect of cryo-stress on the cellular ultrastructure of the shoot tip of the WT and the *wrky22.1* mutant. (A) Schematic view of the shoot tip, showing the first three layers of the apical dome. (B–M) Transmission electron micrographs of meristematic cells visualized after phases I–III. (B–G) WT, (H–M) *wrky22.1*. CW, cell wall; M, mitochondrion; N, nucleus; P, plastid; PG, plastoglobulus; V, vacuole.

Ultrastructure analysis of meristematic (Fig. 4) cells revealed that cell size and vacuoles appear to be reduced as an effect of PVS2 treatment (phase II) (Fig. 4C). Cell organelles, most pronounced for plastids and mitochondria, either increased in size or began to degrade, characterized by formation of plastoglobuli (Fig. 4F). In comparison, WT cells of phase I (Fig. 4B, E) and phase III (Fig. 4D, G) look similar. Nuclei and vacuoles of the cells appeared prominent and the cytoplasm of cells was homogeneous and even structured. During the recovery period (phase III), all symptoms associated with dehydration and organelle degradation disappeared (Fig. 4D, G). The ultrastructure of the *wrky22.1* mutant tissue in all three phases was indistinguishable from that of the WT cells (Fig. 4H–M).

### RNA processing acts as a key regulator during cryoprotectant treatment

**Fig. 5.**
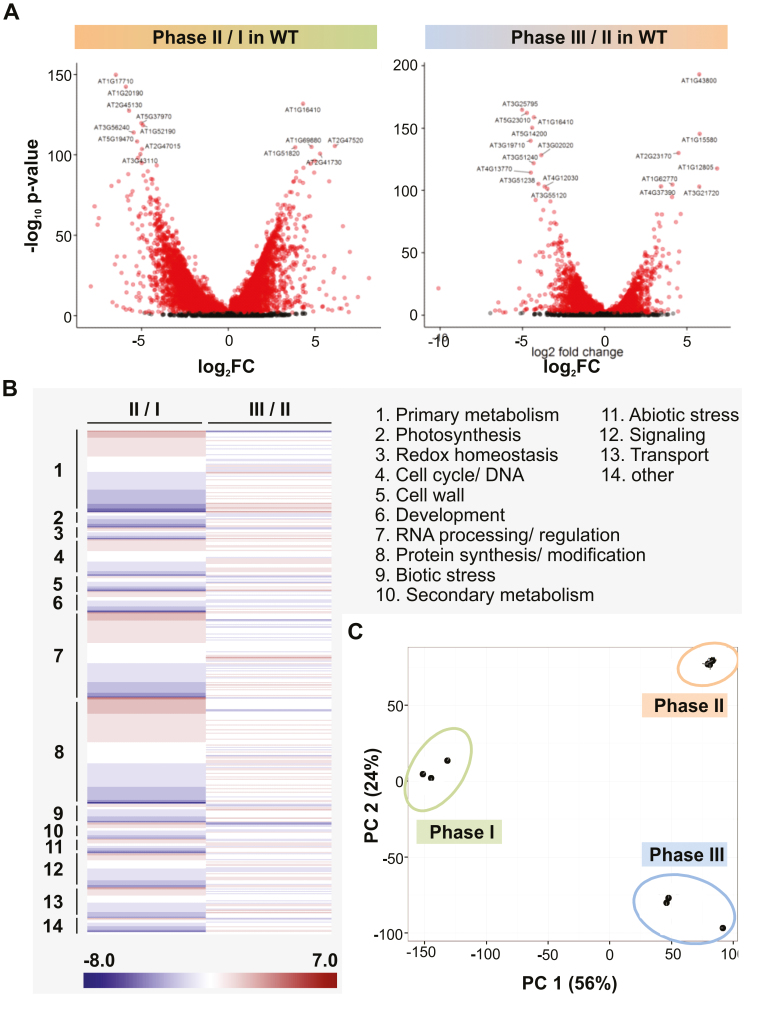
The transcriptome of WT shoot tip explants at the end of phases I–III. (A) The analysis identified 12 067 genes as changed with respect to their transcript abundance between phases I and II, and 6349 between phases II and III (*P*-value <0.01 after multiple hypothesis correction). Genes associated with a *P* value <10^–100^ are labeled with their AGI code. (B) The MapMan bins of the DEGs identified in the contrasts phases II versus I and III versus II. Red indicates increased abundance and blue decreased abundance, with the color intensity reflecting the fold of differential gene expression. (C) A principal component analysis confirms the difference between the three phases.

**Fig. 6.**
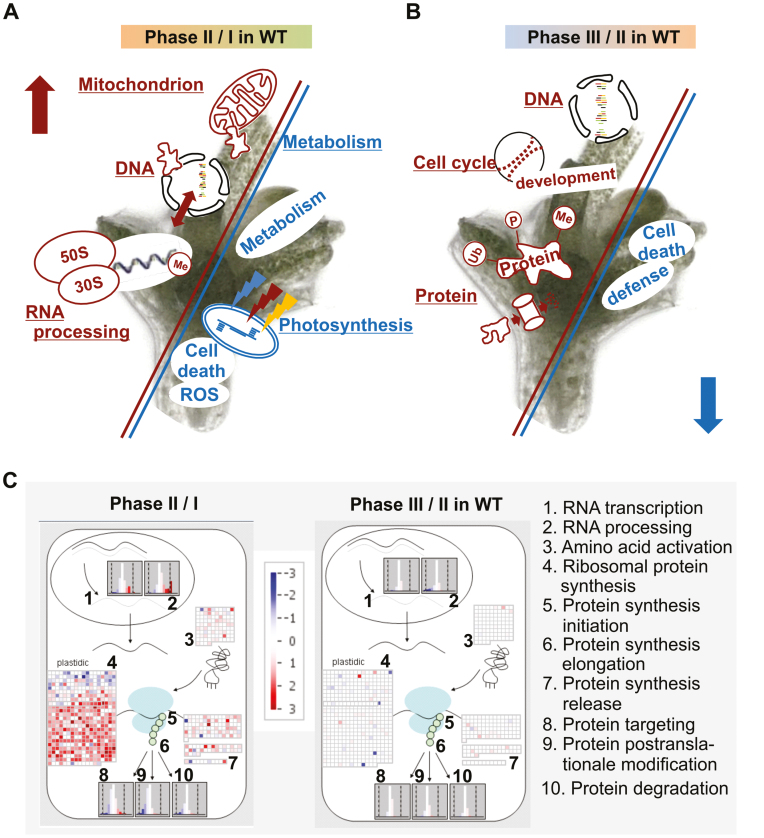
GO term enrichment and MapMan functional assignment of the WT transcriptome. Enriched GO terms among the regulated genes in the contrast (A) phase II versus I and (B) phase III versus II; enriched GO terms among genes with higher abundance are shown in red and among lower abundance in blue. (C) MapMan mapping of RNA–protein synthesis. Each square represents the transcription of a single gene within a given pathway. Hochberg-corrected transcripts with higher abundance are shown in red, and lower abundance in blue. The color intensity reflects the fold of differential gene expression.

The comparison between the transcriptomes of the WT explants sampled in phases I and II identified 12 067 DEGs, and that between phases II and III 6349 DEGs (Fig. 5A). The two sets of DEGs were assigned to MapMan bins, as depicted in Fig. 5B. A principal component analysis showed a clear separation between the three phases (Fig. 5C). The GO enrichment analysis established that the PVS2 treatment had a major positive impact on the abundance of transcript generated by genes encoding proteins involved in RNA processing and methylation, mitochondrial processes, DNA modification, and nuclear targeting, while the major classes of genes negatively impacted were related to photosynthesis (in particular the light response and chlorophyll synthesis) as well as the metabolism of saccharides, lipids, fatty acids, and amino acids (Fig. 6A; Supplementary Dataset 1). The MapMan analysis confirmed the conclusions drawn from the GO term enrichment analysis: transcripts encoding proteins involved in ribosomal protein synthesis became notably more abundant in phase II than in phase I, but this difference was not apparent between phases II and III (Fig. 6C; Supplementary Table S2). Genes encoding components of RNA processing and ribosomal protein synthesis, as well as metabolism and photosynthesis, were represented in the set of the most highly up- and down-regulated genes (Tables 1, 2; Supplementary Dataset 2). The transcriptomic data-based conclusions were validated for the three selected RNA processing genes *NOP56* (*At1g56110*), *NOP58* (*At3g05060*), and *EBP2* (*At3g22660*) using qRT–PCR: in each case, transcript abundance was boosted by the PVS2 treatment (Supplementary Fig. S2).

**Table 1.** The 50 most highly up-regulated genes in the WT explants identified in the contrast phase II versus I

	Locus	FC	Gene name	Mapman functional description
1*	**AT2G47520**	**6.1**	*HYPOXIA RESPONSIVE (ERF) 2 (HRE2)*	**RNA.regulation of transcription**
2	AT4G12490	6.1	*AZI3*	misc.protease inhibitor
3	AT3G46280	5.0	*kinase-like protein*	signalling.receptor kinases
4	AT2G26150	4.9	*HEAT SHOCK TRANSCRIPTION FACTOR A2*	stress.abiotic.heat
**5**	**AT1G10585**	**4.9**	*basic helix-loop-helix DNA-binding superfamily protein*	**RNA.regulation of transcription**
6	AT1G69880	4.8	*THIOREDOXIN H-TYPE 8*	redox.thioredoxin
**7**	**AT5G59240**	**4.7**	*Ribosomal protein S8e family protein*	**protein.synthesis.ribosomal protein**
**8**	**AT3G17609**	**4.7**	*HY5-HOMOLOG*	**RNA.regulation of transcription**
9	AT4G22470	4.6	*Protease inhibitor/lipid-transfer protein*	misc.protease inhibitor
**10**	**AT5G51440**	**4.5**	*HSP20-like chaperones superfamily protein*	**stress.abiotic.heat**
**11**	**AT1G05680**	**4.4**	*UGT74E2*	**hormone metabolism.salicylic acid**
12*	**AT4G06746**	**4.4**	*RELATED TO AP2 9* (*RAP2.9)*	**RNA.regulation of transcription**
13**	AT3G51240	4.4	*FLAVANONE 3-HYDROXYLASE*	secondary metabolism.flavonoids
**14**	**AT1G64220**	**4.4**	*TRANSLOCASE OF OUTER MEMBRANE 7-2*	**transport mitochondrial membrane**
15	AT1G17180	4.4	*GLUTATHIONE S-TRANSFERASE TAU 25*	misc.glutathione S transferases
**16**	**AT3G09680**	**4.3**	*Ribosomal protein S12/S23 family protein*	**protein.synthesis.ribosomal protein**
17**	AT1G16410	4.3	*CYTOCHROME P450 79F*	secondary metabolism.sulfur-containing
18	AT2G16060	4.1	*HEMOGLOBIN 1*	redox.heme
19**	AT5G13930	4.1	*CHALCONE SYNTHASE*	secondary metabolism.flavonoids
**20**	**AT5G14200**	**4.0**	*ISOPROPYLMALATE DEHYDROGENASE 1*	**amino acid metabolism.synthesis**
**21**	**AT5G40040**	**4.0**	*60S acidic ribosomal protein family*	**protein.synthesis.ribosomal protein**
22	AT5G39580	4.0	*Peroxidase superfamily protein*	misc.peroxidases
23	AT2G15620	3.9	*NITRITE REDUCTASE 1*	N-metabolism.nitrate metabolism
**24**	**AT3G19710**	**3.9**	*BRANCHED-CHAIN AMINOTRANSFERASE4*	**amino acid metabolism.synthesis**
**25**	**AT3G12860**	**3.9**	*NOP56-like pre RNA processing ribonucleoprotein*	**protein.synthesis.ribosome biogenesis**
26	AT5G41670	3.9	*6-phosphogluconate dehydrogenase family protein*	OPP.oxidative
27	AT3G46230	3.9	*HSP17.4*	stress.abiotic.heat
28**	AT5G07990	3.9	*CYTOCHROME P450 75B1*	secondary metabolism.flavonoids
**29**	**AT1G32880**	**3.9**	*ARM repeat superfamily protein*	**protein.targeting.nucleus**
30	AT1G51820	3.9	*Leucine-rich repeat protein kinase family protein*	signalling.receptor kinases.misc
**31**	**AT1G58684**	**3.8**	*Ribosomal protein S5 family protein*	**protein.synthesis.ribosomal protein**
**32**	**AT1G58983**	**3.8**	*Ribosomal protein S5 family protein*	**protein.synthesis.ribosomal protein**
33	AT1G02820	3.8	*LEA3*	development
**34**	**AT3G06900**	**3.8**	*U4 SMALL NUCLEOLAR RNA2*	**RNA.processing**
35	AT5G40850	3.8	*UROPHORPHYRIN METHYLASE 1*	tetrapyrrole synthesis
36	AT1G51850	3.8	*Leucine-rich repeat protein kinase family protein*	signalling.receptor kinases.misc
37	AT4G33070	3.7	*ATPDC1*	fermentation.PDC
38	AT1G24280	3.7	*G6PD3*	OPP.oxidative
39**	AT5G23010	3.7	*2-ISOPROPYLMALATE SYNTHASE 3*	secondary metabolism.sulfur-containing
40	AT4G12480	3.7	*PEARLI 1*	misc.protease inhibitor
**41**	**AT1G23410**	**3.7**	*Ribosomal protein S27a*	**protein.synthesis.ribosomal protein**
42	AT4G12500	3.6	*Bifunctional inhibitor/lipid-transfer protein*	misc.protease inhibitor
43	AT1G78050	3.6	*PGM*	glycolysis.unclear
44	AT1G14120	3.6	*AUXIN OXIDASE*	misc.oxidases
**45**	**AT5G13490**	**3.6**	*ADP/ATP CARRIER 2*	**transport.unspecified cations**
**46**	**AT3G02020**	**3.5**	*ASPARTATE KINASE 3*	**amino acid metabolism**
47	AT2G03230	3.5	*GCK domain-containing protein*	not assigned.unknown
**48**	**AT4G25630**	**3.5**	*FIBRILLARIN 2*	**protein.synthesis.ribosome biogenesis**
**49**	**AT5G27120**	**3.5**	*NOP58-like pre RNA processing ribonucleoprotein*	**RNA.regulation of transcription**
**50**	**AT5G53290**	**3.5**	*CYTOKININ RESPONSE FACTOR 3*	**RNA.regulation of transcription**

MapMan bins consistent with GO term enrichment are shown in bold. Genes labeled with an asterisk have been associated in the literature with either the drought stress response (*) or products of secondary metabolism (**).

**Table 2.** The 50 most highly down-regulated genes in the WT explants identified in the contrast phase II versus I

	Locus	FC	Gene Name	Mapman Functional Description
**1**	**AT2G33830**	**–7.7**	*DORMANCY ASSOCIATED GENE 2*	**hormone metabolism**
2	AT1G31580	–7.6	*ECS1*	stress.biotic
**3**	**AT1G56600**	**–7.5**	*GALACTINOL SYNTHASE 2*	**minor CHO metabolism**
4*	AT1G20440	–6.5	*COLD-REGULATED 47*	stress.abiotic.unspecified
5	AT1G17710	–6.5	*Pyridoxal phosphate phosphatase-related*	misc.acid and other phosphatases
6	AT1G26945	–6.4	*PACLOBUTRAZOL RESISTANCE 6*	not assigned.unknown
7	AT5G45890	–6.2	*SENESCENCE-ASSOCIATED GENE 12*	protein.degradation
8	AT1G20190	–5.9	*EXPANSIN 11*	cell wall.modification
9*	AT1G29395	–5.9	*COLD REGULATED 314 INNER MEMBRANE 1*	not assigned.no ontology
10	AT1G52690	–5.9	*LATE EMBRYOGENESIS ABUNDANT*	development
11	AT3G09922	–5.8	*INDUCED BY PHOSPHATE STARVATION1*	not assigned.unknown
12	AT1G56220	–5.8	*Dormancy/auxin associated family protein*	development.unspecified
13	AT2G45130	–5.7	*SPX DOMAIN GENE 3*	stress.abiotic
14*	AT1G73330	–5.7	*DROUGHT-REPRESSED 4*	stress.biotic
**15**	**AT3G27690**	**–5.6**	*LHCB2.3*	**PS.lightreaction.photosystem II**
16	AT5G24490	–5.6	*30S ribosomal protein*	protein.synthesis.ribosomal protein
**17**	**AT3G01500**	**–5.5**	*SALICYLIC ACID-BINDING PROTEIN 3*	**TCA/org transformation**
**18**	**AT3G15450**	**–5.5**	*Aluminium induced protein*	**hormone metabolism**
19	AT3G56240	–5.5	*COPPER CHAPERONE*	metal handling
20	AT5G14565	–5.3	*MICRORNA398C*	micro RNA, natural antisense
**21**	**AT1G09350**	**–5.3**	*GALACTINOL SYNTHASE 3*	**minor CHO metabolism**
**22**	**AT5G19470**	**–5.3**	*NUDIX HYDROLASE HOMOLOG 24*	**nucleotide metabolism**
**23**	**AT3G26180**	**–5.2**	*CYP71B20*	**misc.cytochrome P450**
**24**	**AT3G02040**	**–5.2**	*SENESCENCE-RELATED GENE 3*	**lipid metabolism**
25*	AT1G20450	–5.2	*EARLY RESPONSIVE TO DEHYDRATION 10*	stress.abiotic.unspecified
26	AT2G41870	–5.2	*Remorin family protein*	RNA.regulation of transcription
27	AT3G16670	–5.2	*Pollen Ole e 1 allergen*	not assigned.unknown
**28**	**AT3G26740**	**–5.2**	*CCR-LIKE*	**signalling.light**
**29**	**AT1G80920**	**–5.2**	*TOC12*	**stress.abiotic.heat**
30	AT5G06760	–5.1	*LATE EMBRYOGENESIS ABUNDANT 4–5*	development
**31**	**AT3G62550**	**–5.1**	*Adenine nucleotide alpha hydrolases-like protein*	**hormone metabolism**
32	AT3G55240	–5.1	*Protein coding*	not assigned.unknown
33	AT5G37970	–5.0	*S-adenosyl-L-methionine-dependent methyltransferases superfamily protein*	**hormone metabolism.salicylic acid**
34*	AT4G25490	–5.0	*DRE BINDING PROTEIN 1B (CBF1)*	RNA.regulation of transcription
**35**	**AT3G63210**	**–5.0**	*MEDIATOR OF ABA-REGULATED DORMANCY 1*	**hormone metabolism**
36	AT2G47015	–5.0	*MICRORNA408*	micro RNA, natural antisense
37	AT1G75380	–5.0	*BIFUNCTIONAL NUCLEASE IN BASAL DEFENSE RESPONSE 1*	stress.abiotic.touch/wounding
38	AT1G67265	–4.9	*ROTUNDIFOLIA LIKE 21*	development.unspecified
39	AT1G52190	–4.9	*NITRATE TRANSPORTER 1.11*	transport.peptides and oligopeptides
40	AT5G49360	–4.9	*BETA-XYLOSIDASE 1*	cell wall.degradation
41	AT1G01470	–4.9	*LATE EMBRYOGENESIS ABUNDANT 14*	development
**42**	**AT1G23730**	**–4.9**	*BETA CARBONIC ANHYDRASE 3*	**TCA/org transformation**
**43**	**AT1G79040**	**–4.9**	*PHOTOSYSTEM II SUBUNIT R*	**PS.lightreaction.photosystem II**
44	AT1G18870	–4.9	*ISOCHORISMATE SYNTHASE 2*	Co-factor and vitamine metabolism
45	AT2G17040	–4.9	*NAC DOMAIN CONTAINING PROTEIN 36*	development.unspecified
**46**	**AT1G20620**	**–4.8**	*CATALASE 3*	**redox.dismutases and catalases**
47	AT1G73540	–4.8	*NUDIX HYDROLASE HOMOLOG 21*	nucleotide metabolism
48	AT5G39520	–4.8	*hypothetical protein*	not assigned.unknown
**49**	**AT1G11530**	**–4.7**	*ATCXXS1*	**redox.thioredoxin**
50	AT1G28330	–4.7	*DORMANCY-ASSOCIATED PROTEIN 1*	development.unspecified

MapMan bins consistent with GO term enrichment are shown in bold. Genes labeled with an asterisk have been associated in the literature with either the drought stress response (*) or products of secondary metabolism (**)

The DEGs identified included five genes known to be inducible by drought stress [*ERF2* (*At4g06746*), *RAP2.9* (*At2g47520*), *DR4* (*At1g73330*), *ERD10* (*At1g20450*), and *CBF1* (*At4g25490*)], along with two low temperature stress-inducible genes [*COR47* (*At1g20440*) and *COR413IM1* (*At1g29395*)]: these are marked by a single asterisk in Tables 1 and 2. The set of genes with higher abundance included a number associated with the defense response, in particular related to products of secondary metabolism (marked with a double asterisk in Table 1).

A GO term enrichment analysis of the set of DEGs in phase III indicated that genes with higher abundance could be assigned to terms of development, cell cycling, protein modification/ubiquitination, and DNA modification/replication, while apoptosis and defense were suppressed (Fig. 6B; Supplementary Dataset S1). Consistent with MapMan analysis, genes showing higher abundance were prominently related to auxin-mediated cell growth, and those with lower abundance to products of secondary metabolism (Supplementary Fig. S3).

### Cryoprotectant treatment affects genes relevant for photosynthesis in the *wrky22* mutant shoot tip explants

**Fig. 7.**
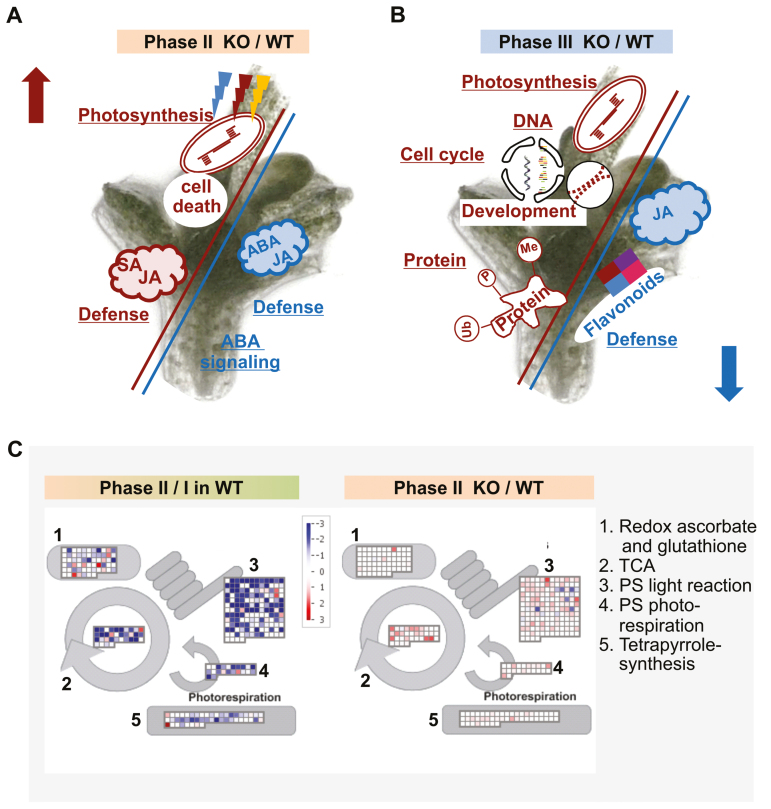
GO term enrichment and MapMan functional assignment of the *wrky22* mutant transcriptome. Enriched GO terms among the regulated genes in the contrast (A) phase II and (B) phase III in the *wrky22.1* mutant over the WT. Enriched GO terms among genes with higher abundance are shown in red and with lower abundance in blue. (C) MapMan mapping of primary metabolism. Each square represents the transcription of a single gene within a given pathway. Hochberg-corrected transcripts with higher abundance are shown in red, and lower abundance in blue. The color intensity reflects the fold of differential gene expression. TCA, tricarboxylic acid cycle.

In all, 124 genes were differentially transcribed between the WT and the *wrky22* mutant when the explants were sampled at the end of phase I, as were 2599 at the end of phase II and 1119 at the end of phase III. A principal component analysis clearly distinguished the three phases, and highlighted the genotypic difference between the WT and the *wrky22* mutant in phases II and III (Supplementary Fig. S4). Effects of *WRKY22* were marginal during shoot tip preparation. The most over-represented category of DEGs was associated with secondary cell wall synthesis, in particular genes encoding PROLINE-RICH EXTENSIN-LIKE FAMILY PROTEINS (Supplementary Datasets S1, S2). These findings suggested that the *wrky22* mutant shoot tips were compromised with respect to the strength of their secondary cell walls. Transcriptome changes associated with cryoprotectant treatment in phase II of the *wrky22* mutant explants showed the high importance of photosynthesis and subsequently an adapted response to light, scavenging of reactive oxygen species (ROS), and apoptosis. The MapMan analysis confirmed the importance of photosynthesis during phase II. Transcripts relevant for PSI and PSII and the Calvin cycle (Fig. 7C; Supplementary Fig. S5) seemed to be key aspects in the *wrky22* mutant acclimation response. These findings were validated for the three selected genes *RCA* (*At2g39730*), *PSAN* (*At5g64040*), and *RBCS3B* (*At5g38410*) using qRT–PCR. The analysis showed reduced transcript abundance during phase II compared with phase I in both the *wrky22* mutant and the WT explants, and higher transcript expression in the *wrky22* mutant than in the WT during phase II (Supplementary Fig. S2).

Genes encoding the salicylic acid (SA)- and jasmonic acid (JA)-regulated defense response, along with products of secondary metabolism, were over-represented in both the high- and low-abundance categories, while processes relying on ABA signaling were suppressed (Fig. 7A; Supplementary Dataset S1). During phase III, development, DNA/RNA modification, protein modification, and photosynthesis were all promoted in the *wrky22* mutant, while the JA-mediated defense response and flavonoid synthesis were suppressed (Fig. 7B; Supplementary Dataset S1).

### Changes in the mutant transcriptome indicated that *WRKY22* is involved in phytohormone-mediated drought and defense acclimation through crosstalk with assorted transcription factors

**Fig. 8.**
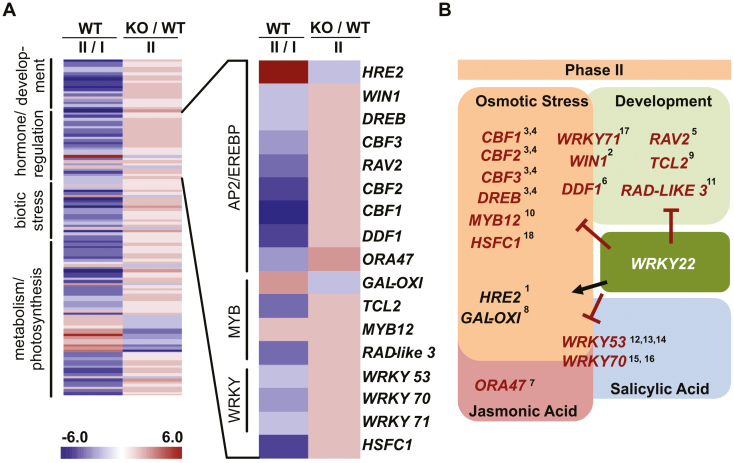
Crosstalk between transcription factors associated with *WRKY22* activity. (A) A heatmap identifying putative interaction partners with *WRKY22* during phase II belonging to the AP2-EREBP, MYB, and WRKY transcription factor family. Genes differentially expressed between phases II and I in WT explants were chosen on the basis of a log_2_ fold change of >1.5, and in the *wrky22.1* mutant explants on the basis of a log_2_ fold change >1.3. Red indicates increased abundance and blue decreased abundance, with the color intensity reflecting the fold of differential gene expression. Genes repressed by *WRK22* are indicated by a red arrow, and those promoted by it by a black arrow. (B) Assignment of function in the context of the biotic and/or abiotic stress response: ^1^(Park *et al.*, 2011), ^2^(Al-Abdallat *et al.*, 2014), ^3^ (Novillo *et al.*, 2004), ^4^(Sakuma *et al.*, 2002), ^5^(Matías-Hernández *et al.*, 2016), ^6^(Kang *et al.*, 2011), ^7^(Chen *et al.*, 2016), ^8^(Loreti *et al.*, 2005), ^9^(Tominaga-Wada *et al.*, 2013), ^10^(Wang *et al.*, 2016), ^11^(Baxter *et al.*, 2007), ^12^(Sun *et al.*, 2003), ^13^(Sun and Yu, 2015), ^14^(Miao and Zentgraf, 2007), ^15^(Li *et al.*, 2013), ^16^(Chen *et al.*, 2017), ^17^(Guo and Qin, 2016), ^18^(Rizhsky *et al.*, 2004).

The set of DEGs (selected on the basis of a log_2_ fold change threshold in transcript abundance of 1.5) between phases I and II for the WT, and between the *wrky22* mutant and the WT during phase II, was assembled to identify potential targets of *WRKY22.* Of these, 145 were assigned to MapMan bins associated with development, hormone and transcript regulation, biotic stress, and photosynthesis (Fig. 8A). The group of DEGs assigned to the hormone and regulation category included a number of members of the AP2-EREBP, MYB, and WRKY TF families. Four of these [*WRKY71* (*At1g29860*), *WIN1* (*At5g11190*), *WRKY53* (*At4g23810*), and *WRKY70* (*At3g56400*) are known to be inducible by more than one stress agent. The products of certain dehydration-responsive binding protein/C-repeat binding factor (CBF)-encoding genes, as well as those of *DDF 1* (*At1g12610*), *HRE2* (*At2g47520*), and *GAL-OXI* (*At3g27220*) are known to be involved in the regulation of the osmotic stress response, while those of *RAV2* (*At1g68840*), *TCL2* (*At2g30424*), and *RAD-LIKE3* (*At4g36570*) control two or more developmental processes; finally, the product of *ORA47* (*At1g74930*) was identified as acting in the JA-regulated defense response (see references in Fig. 8B). The presence of *WRKY22* resulted in the suppression of most of these genes (the exceptions were *GAL-OXI* and *HRE2*), which supported the existence of crosstalk during osmotic stress acclimation between *WRKY22* and members of the AP2-EREBP, MYB, and WRKY families. Overall, 25 putative interaction partners of *WRKY22* in response to osmotic stress were identified. All of the identified targets, except *RAB18* (*AT5G66400*), contain several *WRKY22*-binding motifs (W-boxes) in their promoter region, confirming a crosstalk between the TFs and target genes (Table 3). *In silico* analysis of the WRKY70 promotor sequence showed a W box (C/T)TGAC(T/C) motif, within the region 1000 bp upstream from the WRKY70 genomic DNA sequence known to interact with WRKY22 (Supplementary Fig. S6A). We were able to validate the transcriptomic data-based conclusions for the WRKY70 (*At3g56400*) TF, using qRT–PCR on Arabidopsis seedlings, which have been exposed to a cryoprotectant and a cooling treatment. In each case, *WRKY70* transcript abundance was increased in the *wrky22.1* and *wrky22.2* T-DNA insertion lines compared with WT seedlings. After PVS2 treatment, a significant increase of *WRKY70* transcript was observed in mutant seedlings (Supplementary Fig. S6B). In contrast to the expression of *WRKY70*, *WRKY53* showed no generally increased transcript abundance in different samples. This may be explained by the fact that whole Arabidopsis seedlings and not only meristem-including shoot tips have been used in the validation experiment.

**Table 3.** DEGs identified in explants from the contrast phase II versus I in the WT, on the basis of a log_2_ fold change threshold of 1.5, and in the phase II *wrky22.1* mutant versus the WT, on the basis of a log_2_ fold change threshold of 1.3

	Locus	WT II/I	KO II	Description	Mapman functional description	Reference
1	AT1G01470	–4.9	1.4	*LATE EMBRYOGENESIS ABUNDANT 14 (LEA14)*	development.late embryogenesis abundant	Jia *et al.* 2014
2	AT1G12610	–3.9	1.7	*DWARF AND DELAYED FLOWERING 1 (DDF1)*	RNA.regulation of transcription.AP2/EREBP	Magome *et al.*, 2008; Kang *et al.*, 2011
3	AT4G25490	–5.0	1.7	*C-repeat/DRE binding factor 1 (CBF1)*	RNA.regulation of transcription.AP2/EREBP	Liu *et al.*, 1998; Sakuma *et al.*, 2002
4	AT4G25470	–4.3	1.7	*C-repeat/DRE binding factor 2 (CBF2)*	RNA.regulation of transcription.AP2/EREBP	Liu *et al.*, 1998; Sakuma *et al.*, 2002
5	AT4G25480	–2.1	1.4	*C-repeat/DRE binding factor 2 (CBF3)*	RNA.regulation of transcription.AP2/EREBP	Liu *et al.*, 1998; Sakuma *et al.*, 2002
6	AT5G21960	–1.6	1.4	*DREB*	RNA.regulation of transcription.AP2/EREBP	Liu *et al.*, 1998; Sakuma *et al.*, 2002
7	AT2G47520	6.1	–1.9	*HYPOXIA RESPONSIVE ETHYLENE RESPONSE FACTOR 2 (HRE2)*	RNA.regulation of transcription.AP2/EREBP	Park *et al.*, 2011
8	AT2G47460	1.8	1.8	*MYB12*	RNA.regulation of transcription.MYB	Wang *et al.*, 2016
9	AT3G27220	2.4	–1.8	*Galactose oxidase/kelch repeat superfamily (GAL-OXI)*	RNA.regulation of transcription.MYB	Loreti *et al* 2005
10	AT3G56400	–2.5	1.3	*WRKY70*	RNA.regulation of transcription.WRKY	Li *et al.*, 2013; Chen *et al.*, 2017
11	AT4G23810	–2.0	1.3	*WRKY53*	RNA.regulation of transcription.WRKY	Sun *et al.*, 2003; Sun and Yu, 2015;
12	AT1G29860	–1.9	1.6	*WRKY71*	RNA.regulation of transcription.WRKY	Guo and Quin, 2016
13	AT4G11650	–4.2	1.3	*OSMOTIN 34 (OSM34)*	stress.abiotic	Sharma *et al.*, 2013
14	AT3G24520	–3.8	1.4	*Heat shock transcription factor C1 (HSFC1)*	RNA.regulation.transcription.HSF	Rizhsky *et al* 2004
15	AT1G20440	–6.5	2.2	*COLD REGULATED 47 (COR47)*	stress.abiotic.unspecified	Wu *et al.*, 2017
16	AT1G20450	–5.1	1.6	*EARLY RESPONSIVE TO DEHYDRATION 10 (ERD10)*	stress.abiotic.unspecified	Wu *et al.*, 2017
17	AT5G66400	–4,5	1.6	*RESPONSIVE TO ABA 18 (RAB18)*	stress.abiotic.unspecified	Wu *et al.*, 2017
18	AT1G73330	–5.6	2.8	*DROUGHT-REPRESSED 4 (DR4)*	stress.biotic.PR-proteins.proteinase inhibitors	Boyce *et al.*, 2003
19	AT3G62410	–4.0	1.5	*CP12 domain-containing protein 2 (CP12-2)*	PS.calvin cycle	López-Calcagno *et al.*, 2017
20	AT3G54050	–3.7	1.5	*HIGH CYCLIC ELECTRON FLOW 1 (HCEF1)*	PS.calvin cycle.FBPase	Soto-Suárez *et al.*, 2016
21	AT1G32060	–4.0	1.5	*PHOSPHORIBULOKINASE (PRK)*	PS.calvin cycle.PRK	López-Calcagno *et al.*, 2017
22	AT2G39730	–4.0	1.6	*RUBISCO ACTIVASE (RCA)*	PS.calvin cycle.rubisco interacting	Zhang *et al.*, 2015
23	AT1G29395	–5.8	1.6	*COLD REGULATED 314 INNER MEMBRANE 1 (COR413IM1)*	not assigned.no ontology	Magome *et al.*, 2008
24	AT1G62480	–4.0	1.3	*Vacuolar calcium-binding protein-related*	signalling.calcium	Boyce *et al.*, 2003
25	AT4G17340	–4.3	1.4	*tonoplast intrinsic protein 2;2 (TIP2;2)*	transport.Major Intrinsic Proteins.TIP	Zhu *et al.*, 2014

Gene functions are assigned either by MapMan analysis or from the literature in the context of the regulation of osmotic stress.

### Stomatal closure induced by PVS2 treatment differed between the WT and the *wrky22* mutants

**Fig. 9.**
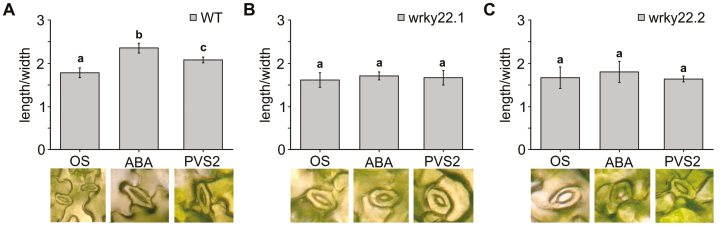
Changes in stomatal aperture induced by *WRKY22* in the presence of ABA and PVS2. (A) WT, (B) *wrky22.1* mutant, (C) *wrky22.2* mutant. The stomatal aperture ratio (length/width) was calculated from 80 stomata in three biological replicates; SD (*n*=3). Statistical significance was calculated using one-way ANOVA followed by Holm–Sidak post-hoc test. Mean values marked by the same letter did not differ significantly from one another (*P*≤0.001).

To reveal the function of *WRKY22* in stomatal movement, leaves of both the WT and the two independent *wrky22* mutants were treated with either ABA or PVS2. Both treatments promoted the closure of guard cells in WT leaves (Fig. 9A) but not in those of either mutant (Fig. 9B,C), which implied that the loss of WRKY22 function induced a greater level of sensitivity to osmotic stress. A follow-up experiment, in which water was withheld from both WT and *wrky22* mutant plants for 18 d, confirmed that the loss of *WRKY22* function significantly reduced the plants’ FW and the rosette size (Supplementary Fig. S7).

## Discussion

Achieving a high level of post-cryogenic viability is important to preserve currently endangered plant species and maintain biodiversity *ex situ*. This requires explants to have the capacity to properly respond to a variety of stresses, which include wounding and the exposure to osmotic, chemical, and low temperature stress.

**Fig. 10.**
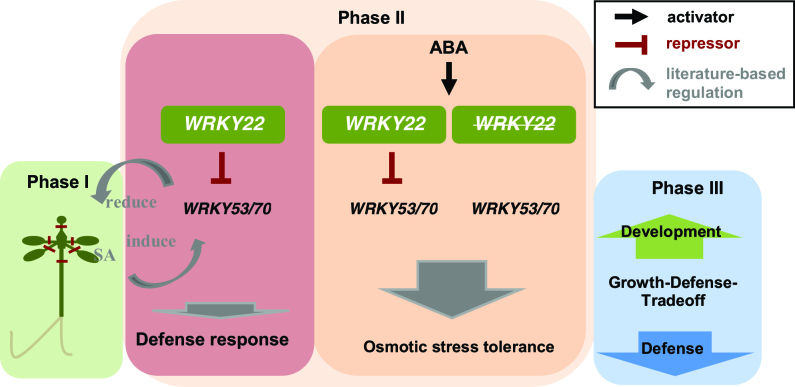
A proposed function for *WRKY22* during the cryopreservation process. *WRKY22* suppresses the transcription of *WRKY53* and *WRKY70*, resulting in an altered salicylic acid (SA)-mediated wounding response and an altered osmotic stress response as suggested by the changed stomatal opening behavior. The open stomata phenotype exhibited by the *wrky22.1* mutant results in a greater volume of H_2_O loss and CO_2_ fixation, and a change to the chloroplasts’ capacity. A higher energy demand may limit the trade-off between growth and defense, resulting in the mutant explants suffering a compromised level of post-cryopreservation recovery.

This study showed that Arabidopsis WT shoot tips could overcome cryo-induced stress response accompanied by high post-cryogenic recovery. A transcriptomic and modeling (Fig. 10) approach and further molecular characterization of WT and T-DNA insertion plants unraveled the molecular mechanisms underlying cryopreservation after shoot tip preparation (phase I), cryoprotectant treatment (phase II), and first day of recovery (phase III).

Cryoprotectant treatment initiated a number of changes to *A. thaliana* WT shoot tips, including the induction of a more de-differentiated status (Fig. 4) and an increase in protein synthesis (Fig. 6). At the same time, a limited response to apoptosis, ROS production, and photosynthesis promoted processes related to development, cell cycling, and protein turnover in phase III.

Moreover, this study provided significant insight into the basic function of WRKY22 and into processes in which multiple abiotic stressors act simultaneously. The response of the *wrky22* mutant was rather different compared with the WT, comprising a less organized stress response, specifically related to phytohormone-mediated processes and photosynthesis. This response was more permissive of damage, resulting in a significant proportion of the rewarmed explants being able to form only non-differentiated callus rather than new shoot material. The loss of the functional *WRKY22* transcript limited the responsiveness to phytohormone-mediated defense (SA and JA) and drought stress (ABA) responses in mutant shoot tips in phase I and phase II.

### The WT explant’s transcriptome response to cryo-stressors

It has been proposed that recovery post-cryopreservation is largely compromised by a build-up of ROS, together with a reduced capacity to produce the detoxifying antioxidant enzymes (Uchendu *et al.*, 2010; Chen *et al.*, 2015; Ren *et al.*, 2015; Gross *et al.*, 2017). The transcript abundance of the redox stress marker genes *ATH8* (*At1g69880*), *AHB1* (*At2g16060*), *APX2* (*At3g09640*), and *GPX7* (*At4g31870*) was increased by the cryoprotectant treatment, but many other known ROS marker genes were down-regulated in the WT explants, and only marginally altered in the *wrky22* mutant. To further address the role of ROS during cryopreservation, additional biochemical investigations will be needed.

The PVS2 reagent combines a number of different cryoprotectant substances, some of which are potentially toxic for meristematic cells (Volk *et al.*, 2006). One of the PVS2-intrinsic cryoprotectants is DMSO; given that this compound inhibits electron transport in the chloroplast (Reeves and Hall, 1977), it can be expected to affect the transcription of genes associated with photosynthesis. To reduce its chemotoxicity, the cryoprotectant treatment is typically conducted in the dark at a low temperature, conditions which suppress photosynthesis. Osmotic stress, resulting from the partial dehydration of the explant, is required to avoid the formation of ice crystals during the cooling step. The ultrastructural analysis showed typical cellular stress indicators such as less dense appearing cytoplasm, plasmolysis, formation of plastoglobuli, and an increasing number of small vacuoles. These effects agree with those which have been seen in potato (Kaczmarczyk *et al.*, 2008). It is known that salinity treatment, which imposes osmotic stress, can induce stomatal closure, the inhibition of CO_2_ fixation, and a reduced flux of electrons through PSII (Kilian *et al.*, 2007; Stepien and Johnson, 2009); here, the intensity of transcription in the treated WT explants was reduced with respect to both of the PSII-associated genes *PSBR* (*At1g79040*) and *LHCB2*.*3* (*At3g27690*) (Table 3). Thus, the suggestion is that chemotoxicity and osmotic stress represent significant components of the overall stress imposed by the cryo-stressors.

### The regulatory role of *WRKY22* during cryopreservation

Consistent with what has been reported in the literature (Kloth *et al.*, 2016), changes in *WRKY22* expression initiated transcriptional changes in genes related to the synthesis of the cell wall (Supplementary Dataset S1) and the SA-mediated stress response (Fig. 7A). Most of the changes induced in the transcriptome took place during phase II, possibly reflecting a delayed wounding response. The altered nature of the SA-mediated defense response may have arisen through crosstalk with *WRKY53* and *WRKY70*, both of which are known to act as regulators of SA-mediated gene transcription (Li *et al.*, 2004; Wang *et al.*, 2006; Miao *et al.*, 2007; Kloth *et al.*, 2016). Such crosstalk is supported by the observation that *WRKY70* gene expression is increased in both independent *wrky22* KO mutant lines compared with the WT when the seedlings were exposed to a cold treatment. Since both WRKY53 and WRKY70 have been identified as repressors of stomatal opening (Li *et al.*, 2013; Sun and Yu, 2015), it is tempting to speculate that WRKY22 cooperates with these two TFs in the context of the explants’ acclimation to osmotic stress. WRKY22 might participate in the explants’ acclimation to osmotic stress by impacting on stomata opening–closing control and thereby on the plants’ osmotic stress behavior (Fig. 9). During recovery, a number of genes involved in auxin-driven growth or in histone modification showed higher abundance in the *wrky22* mutant explants, while certain defense response genes were expressed at a lower level (Figs 6B, 7B; Supplementary Dataset S1; Supplementary Fig. S3): this represents a strategy whereby a choice is made between growth and defense (Huot *et al.*, 2014).

The schematic model presented in Fig. 10 summarizes key aspects of the *WRKY22*-mediated regulation of both the osmotic stress and defense responses. The PVS2 treatment and excision of the explant trigger stomatal closure, probably involving *WRKY53* and *WRKY70*, while at the same time the wounding response is orchestrated by genes responding to an SA signal. The putative *wrky22* mutant’s open stomata phenotype would enhance the volume of CO_2_ fixation, driving changes in the transcription of genes encoding PSII at the expense of defense responses necessary during regeneration.

In summary, Arabidopsis represents a suitable model for identifying the mechanistic basis of the response to the combined abiotic stresses imposed by the cryopreservation process. Successful recovery requires a balance between ensuring cellular survival during low temperature storage through de-differentiation and the ability to regenerate a viable plant upon rewarming. Elucidation of the underlying processes is informative to better understand combinatorial stress defense mechanisms in plants.

## Supplementary data

Supplementary data are available at *JXB* online, and access to the transcriptional data set is provided via doi: 10.5447/ipk/2020/6.

Fig. S1. Verification of the inactivated WRKY22 transcript.

Fig. S2. qRT–PCR-selected genes.

Fig. S3. MapMan_Auxin_Secondary Metabolites.

Fig. S4. Principal component analysis of the *wrky22.1* mutant.

Fig. S5. MapMan_Photosynthesis_Wilcoxon Sum Rank.

Fig. S6. qRT–PCR of WRKY70 dependent on Arabidopsis genotype.

Fig. S7. Drought stress experiment.

Dataset S1. GO term enrichment.

Dataset S2. Hitlist 50 UP_DOWN.

Dataset S3. Mothertable RNA-seq reads.

Table S1. Primer list and Arabidopsis genotypes.

Table S2. MapMan_Protein Synthesis_Wilcoxon Sum Rank.

Table S3. Sample preparation for electron microscopy.

eraa224_suppl_Supplementary_File

eraa224_suppl_Supplementary_Dataset_S1

eraa224_suppl_Supplementary_Dataset_S2

eraa224_suppl_Supplementary_Dataset_S3
