# Supplementary material for: The transcription factor WRKY22 is required during cryo-stress acclimation in Arabidopsis shoot tips
Source: J Exp Bot. 2020 Jul 25;71(16):4993–5009. doi: 10.1093/jxb/eraa224 (PMC7475261; doi:10.1093/jxb/eraa224)
Supplement: eraa224_suppl_Supplementary_File [file eraa224_suppl_supplementary_file.pdf]

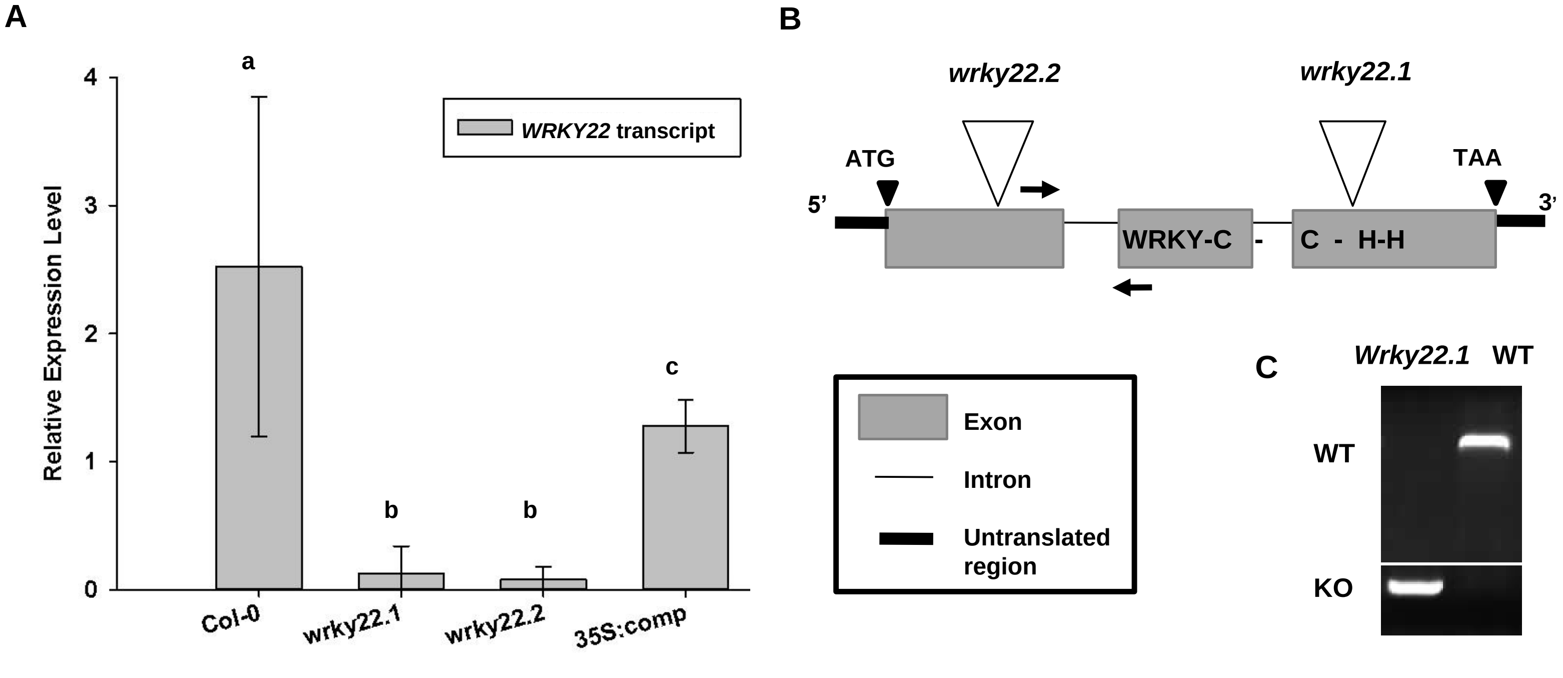

Supplemental Figure S1. Verification of the inactivated *WRKY22* transcript
(A) Quantification of *WRKY22* transcript in Col-0 (WT), *wrky22.1*, *wrky22.2*, and p*35S:comp* by qRT-PCR. Data were normalized to *TIP41 and CLATH*. Error bars indicate ±SD (n=3). Statistical significance was calculated using one-way ANOVA followed by Holm-Sidak post hoc test. Mean values marked by the same letter did not differ significantly from one another ($P \leq 0.05$). (B) Schematic T-DNA insertion sites in *WRKY22*. For *wrky22.1* (SALK_98205), the T-DNA insertion was located in the third exon and for *wrky22.2* (SALK_47120) in the first exon. The highly conserved amino acid sequence of the WRKY domain [WRKYGQK sequence at the N-terminus and a zinc-finger-like motif Cys(2)-His(2) at the C-terminus] was located in the second and third exon. The location of the WRKY domain is indicated as WRKY-C-C-H-H. Arrows show primer combination for qRT-PCR. (C) Homozygous plants were screened on the gDNA level with wildtype (WT) and mutant (KO) specific primer combinations.

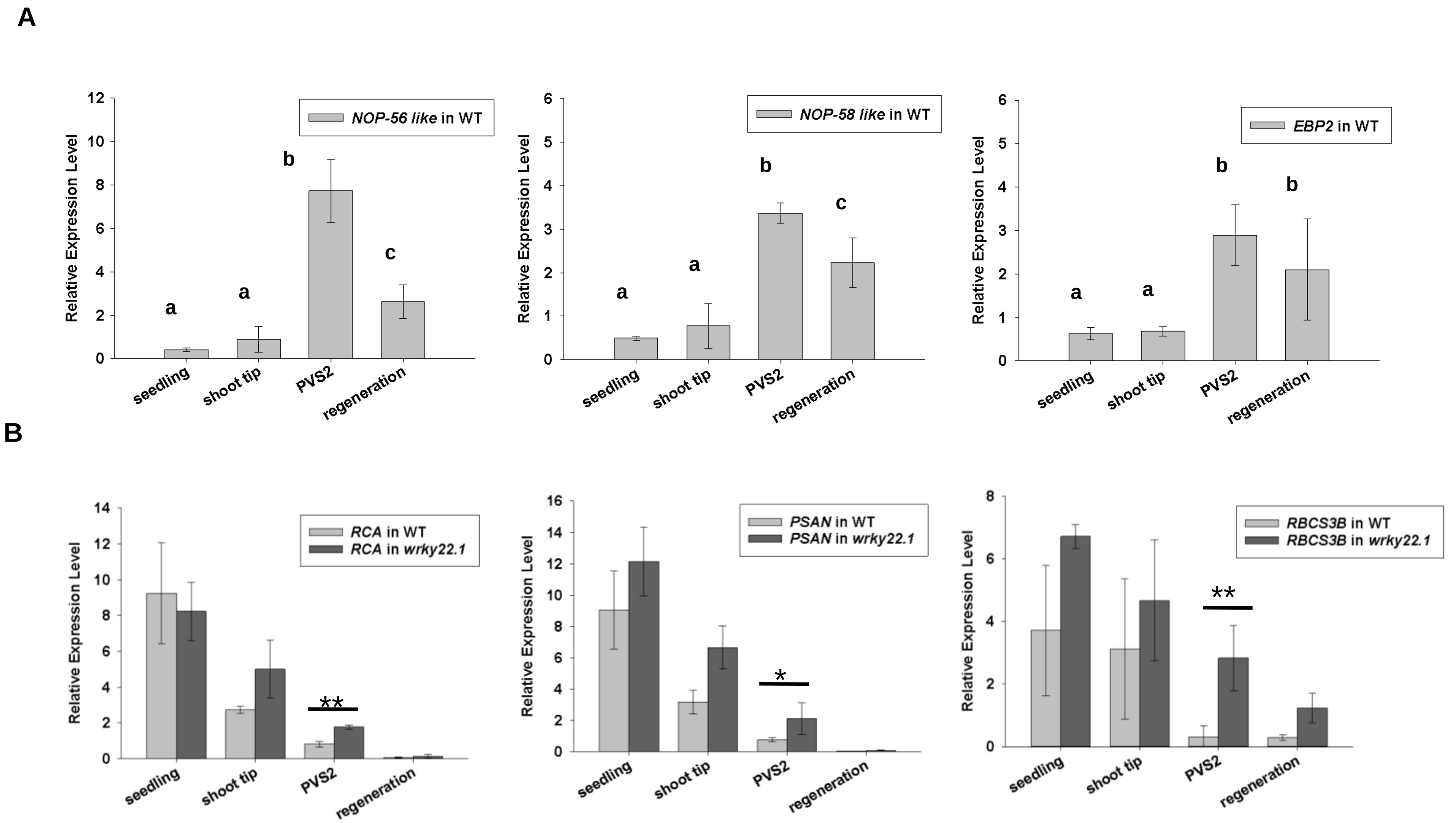

Supplemental Figure S2: (A) Relative transcript expression of *NOP-56 like* (*At3G12860*), *NOP-58-like* (*At3G05060*), and *EBP2* (*At3G22660*) among different stages during cryopreservation. Data were normalized to *TIP41 and CLATH*. Error bars indicate +SD (n=4). Mean values marked by the same letter did not differ significantly from one another (*P*≤0.05) analyzed with one-way ANOVA followed by Holm-Sidac post-hoc. (B) Relative transcript expression of *Rubisco activase* (*RCA*) (*At2G39730*), *PSAN* (*At5G64040*), and *Rubisco small subunit 3B* (*RBCS3B*)(*At5G38410*). Data were normalized to *TIP41 and CLATH*. Error bars indicate +SD (n=4). Statistical differences between WT and *wrky22.1* mutant after PVS2 treatment were calculated using student t-test (* *P*≤0.05; ** *P*≤0.01).

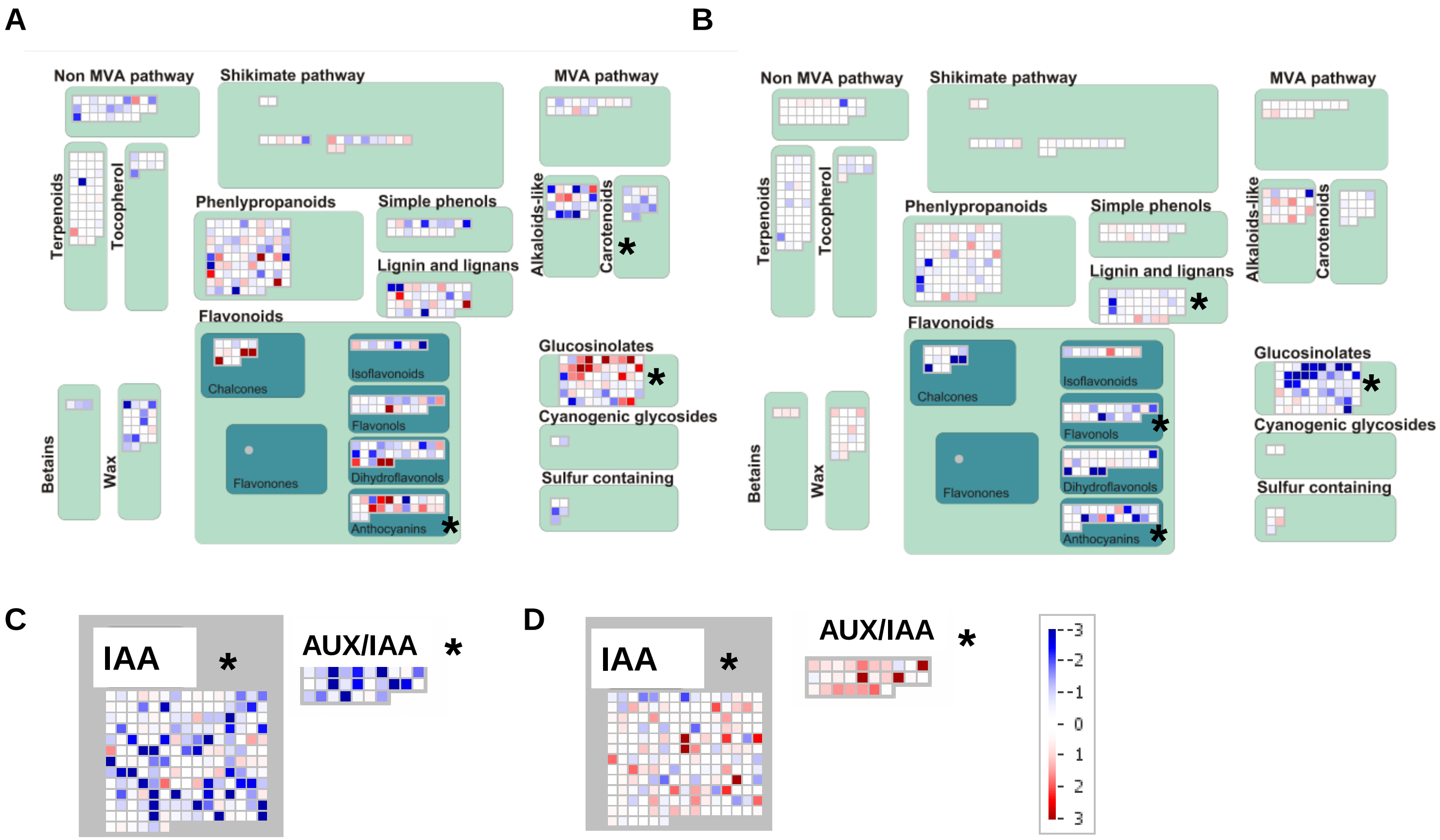

Supplemental Figure S3. MapMan functional assignment of WT transcriptome. MapMan mapping of secondary metabolism in the contrast (A) phase II *vs* I and (B) phase III *vs* II in WT. MapMan mapping of auxin transcript regulation in the contrast (C) phase II *vs* I and (D) phase III *vs* II in WT. Each square represents the transcription of a single gene within a given pathway. Hochberg-corrected transcripts with higher abundance are shown in red, and lower abundance in blue. The color intensity reflects the fold of differential gene expression. *: *P*<0.05 after Benjamini Hochberg correction.

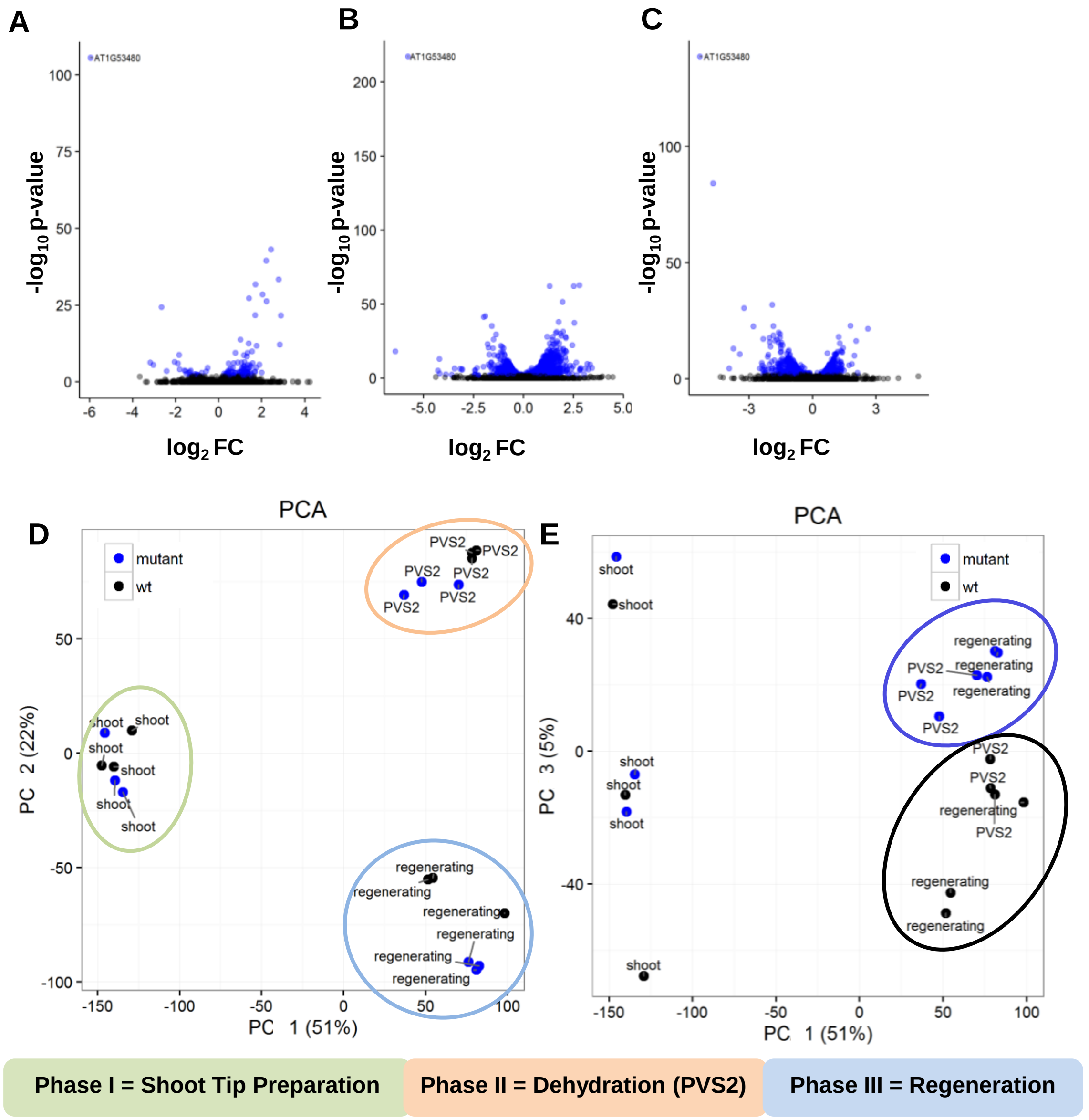

Supplemental Figure S4. The transcriptome of *wrky22.1* mutant shoot tip explants at the end of phase I through III. The analysis identified (A) 123 genes as changed with respect to their transcript abundance in phase I, (B) 2,599 genes in phase II, and (C) 1,119 genes in phase III (p-value < 0.01 after multiple hypothesis correction). Genes associated with a p value $<10^{-100}$ are labeled with their AGI code. (D) A principal component analysis confirms the separation in the first two dimensions according to treatment and (E) genotypic effects in the third dimension.

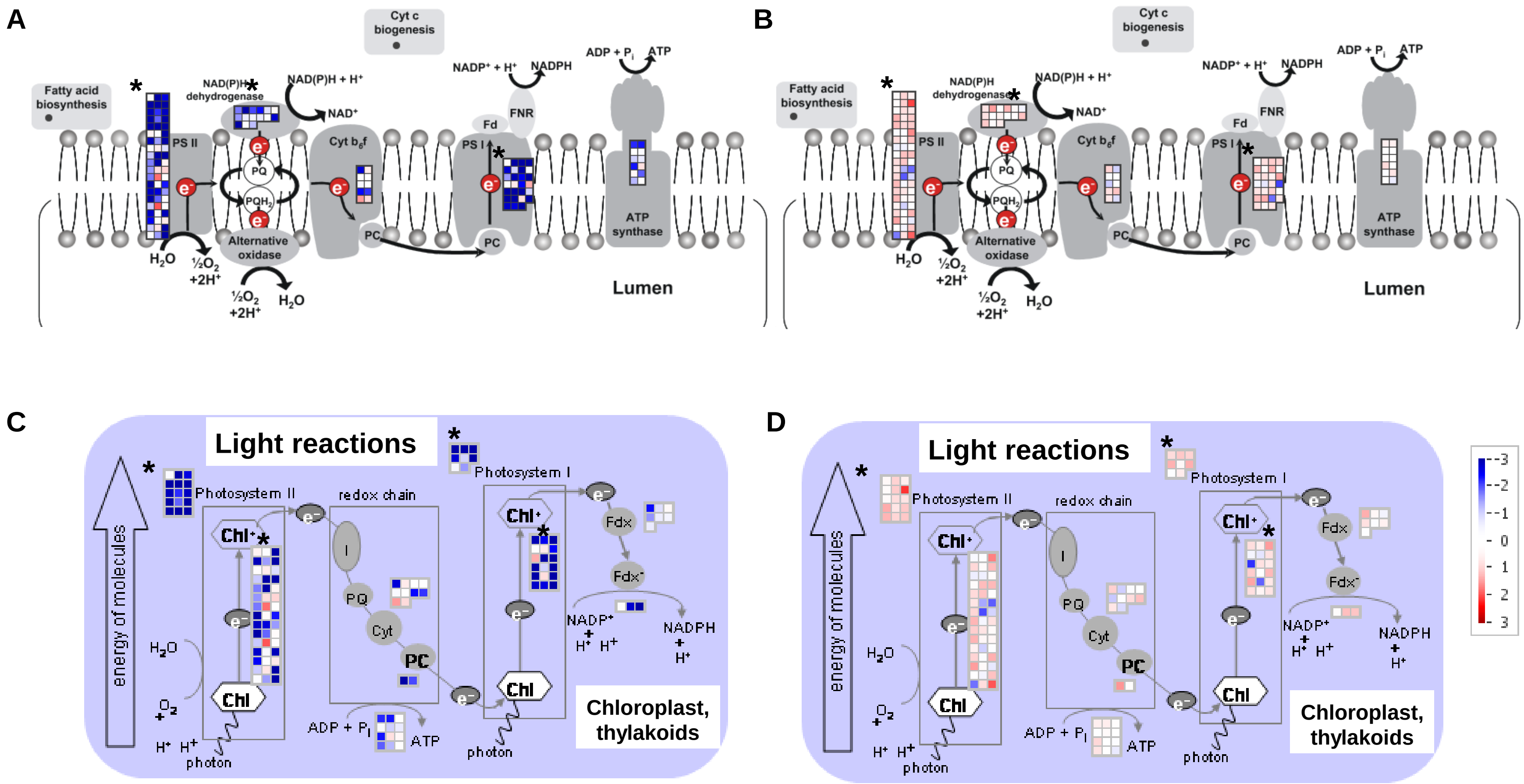

Supplemental Figure S5. MapMan functional assignment of WT and mutant transcriptome. MapMan mapping of photosynthesis in the contrast (A,C) phase II *vs* I and (B,D) WT *vs* mutant in phase II. Each square represents the transcription of a single gene within a given pathway. Hochberg-corrected transcripts with higher abundance are shown in red, and lower abundance in blue. The color intensity reflects the fold of differential gene expression. *: $P<0.05$ after Benjamini Hochberg correction.

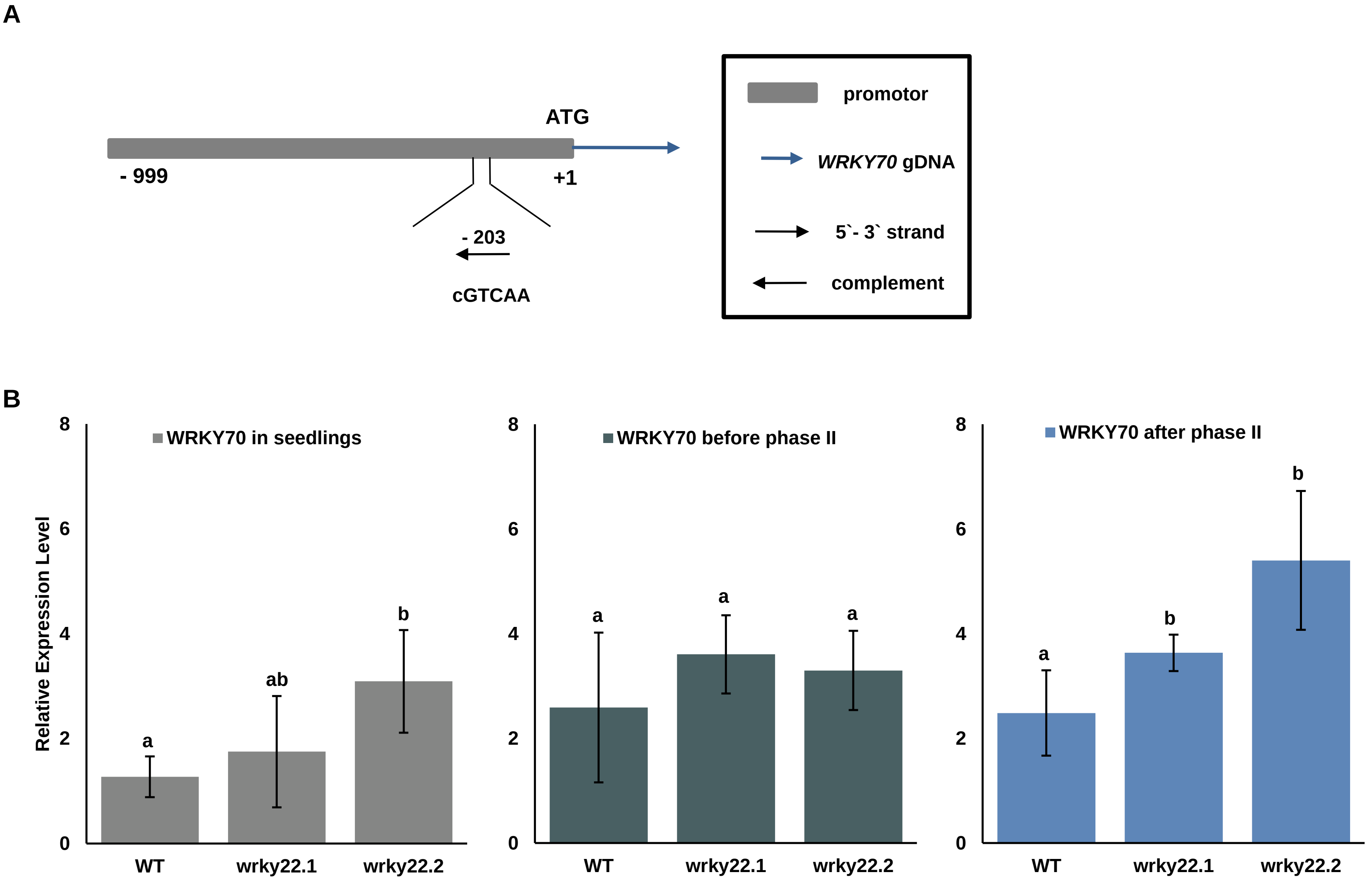

Supplemental Figure S6: *WRKY70* transcript expression is dependent on *Arabidopsis* genotype. (**A**) Scheme of the predicted promotor region 1000 bp upstream of the *WRKY70* gDNA sequence. Putative WRKY22 binding motifs, so called W-box (C/T)TGAC(T/C) element and the position is indicated. (B) Relative transcript expression of *WRKY70* (*At3g56400*) among different stages during cryopreservation. Data were normalized to *TIP41 and CLATH*. Error bars indicate +SD (n=6). Mean values marked by the same letter did not differ significantly from one another (*P*≤0.05) analyzed with one-way ANOVA followed by Holm-Sidac post-hoc.

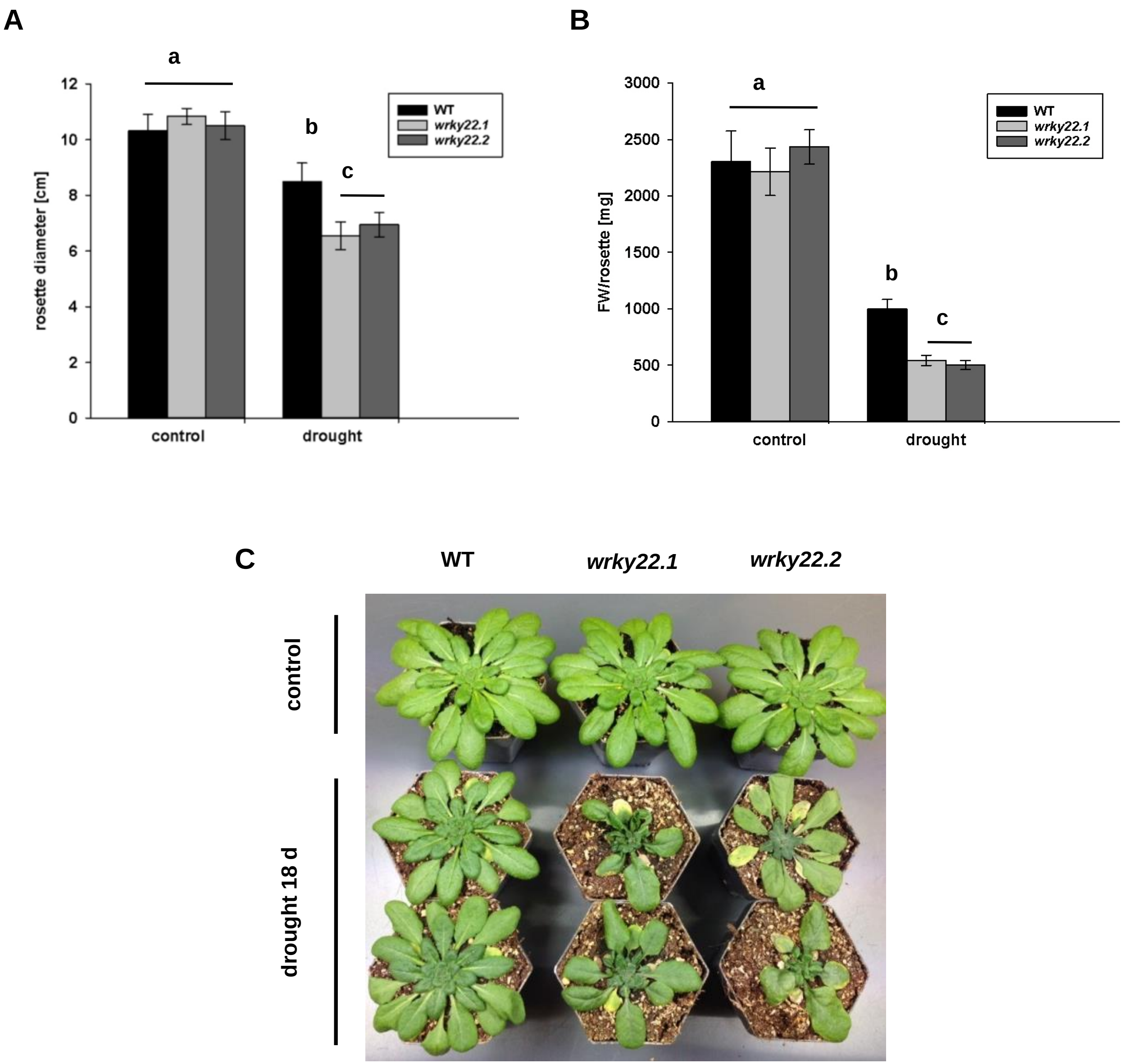

Supplemental Figure S7. Drought stress induced *wrky22* phenotype. Loss of *WRKY22* function reduced (A) rosette diameter (B) fresh weight (FW) and results in a (C) drought stress sensitive phenotype after 18 d without watering (drought). Statistical significance was calculated using one-way ANOVA followed by Holm-Sidak post hoc test. Mean values marked by the same letter did not differ significantly from one another ($P \leq 0.05$).

Supplemantal Table S1 a. Gene specific primers used for tracking of T-DNA insertion lines.

locus	gene	name	sequence *5′ - 3′*	Tm [°C]
At4g01250	*wrky22*	SALK_098205_LP	*fw*-CAC AGA ACC AGA AAC GTC CTC-	59.8
		SALK_098205_RP	*rv*-ATA TTC CTC CGG TGG TAG TGG-	59.8
	wrky22.1	SALK_04720_LP	*fw*-TAC TGC TGA CGG ATT ATT CCG-	57.9
		SALK_04720_RP	*rv*-CCT TTA CCA AAA ATG TAA CGC AG-	57.1
At4g33030	*sqd*	SALK_058117_LP	*fw*-GAA TTG GCC AAT TGG GAT ATC-	55.9
		SALK_058117_RP	*rv*-CAC TTA ACC GGT TCT GTG TGC-	59.8
At3g24500	*mbf1C*	SALK_079508_LP	*fw*-TGT TTC TGG ACC AGG AAC ATC-	57.9
		SALK_079508_RP	*rv*-TTT CTC CAT TTT CGT CTC TGG-	55.9
At1g66180	*asp*	SALK_025595_LP	*fw*-TCC ACA AGT CTT TCA ACA CGA G-	58.4
		SALK_025595_RP	*rv*-GAA CCT TCG CTG AGG GTA ATC-	59.8
	pr5	SALK_055603_LP		
		SALK_055603_RP		
		LBb1.3_BP	ATT TTG CCG ATT TCG GAA C	52.4

Supplemantal Table S1 b. List of quantitative RT-PCR primer sequences

locus	gene	citation	sequence *5′ - 3′*	Tm [°C]
AT5G46630	*Clath*	Czechowski et al., 2005	*fw*-GCC AAT GTT CAC AGC ATC TGG TC-	62,4
			rv-ACC GCT CTT CTC CCA AAC CTT G-	62.1
AT4G34270	*TIP41-like*	Czechowski et al., 2005	*fw*-ATG AAC TGG CTG ACA ATG GAG TG-	60.6
			rv-GAG CTT GGC ATG ACT CTC AC-	59.4
AT4G01250	*WRKY22*	Hsu et al., 2013	*fw*-CGT CCT CTT TCT CTC TCT GCT TCT T-	63.0
			rv-CCA TGC CCA GAC ATC GGA GTT TA	62.4
AT3G12860	*NOP-56 like*		*fw*-AAT CAC CCT CCG GCT ACG G-	61.0
			rv-CTC AGC TCA TCG CTC ATG TAT C-	60.3
AT3G22660	*EBP2*		*fw*-ATG TCA TTG GAA GAG GAT ATA GTA TCA-	60.7
			rv-AGT CCA ATC AAC ATC TTC AGG CCA-	61.0
AT3G05060	*NOP-58 like*		*fw*-CCT ATG AGC TTG GGT CTG TCT-	59.8
			rv-TGA TAT GAT CTT AGC AAG CTC GG-	58.9
AT5G38410	*RBCS3B*		*fw*-CGC AAC AAG TGG ATT CCT TGT-	57.9
			rv-AAT GAG CAG AGA TAA TTC ATA AGA ATG-	57.4
AT2G39730	*RCA*	Zanten et al., 2014	*fw*-TCG TTG AGA GCC TTG GAG TT-	57.3
			rv-CTG AGG TAG GTC TCG GCA A-	58.8
AT5G64040	*PSAN*		*fw*-AAT ACC TCG AGA GGA GCA AAA C-	58.4
			rv-AAA GCA ATA TCT TCT GAG ATA AAT GGA A-	57.8
At3g56400	*WRKY70*	Li et al., 2013	*fw*-CAT GGA TTC CGA AGA TCA CA-	49.7
			rv-CTG GCC ACA CCA ATG ACA A-	51.1

Supplemantal Table S1 c. Primer sequences used for Gateway Cloning

locus	gene	sequence *5′ - 3′*	Tm [°C]
AT4G01250	*WRKY22*	*fw*-CAC CAT GGC CGA CGA TTG-	
		rv-TCA TAT TCC TCC GGT GGT AGT-	

Supplemantal Table S1 d. List of *Arabidopsis* genotypes

Locus	Annotation	T-DNA insertion	mutant
AT4G01250	*WRKY22*	SALK_098205	wrky22.1
		SALK_047120	*wrky22.2*
AT4G33030	*SULFOQUINOVOSYLDIACYL GLYCEROL 1*	SALK_058117C	*sqd*
AT3G24500	*MULTIPROTEIN BRIDGING FACTOR 1C*	SALK_079508	*mbf1C*
AT1G75040	PATHOGENESIS-RELATED GENE 5	SALK_055603	*pr5*
AT1G66180	PUTATIVE *ASPARTYL PROTEASE*	SALK_025595	*asp*

Supplemental Table S2a. Wilcoxon Rank Sum Test followed Benjamini Hochberg correction among changes in transcript expression for MapMan Bins related to RNA-ProteinSynthesis in WT phase II over I (*P*< 0.05).

bin	name	elements	p-value
**27.1**	**RNA.processing**	**379**	**< 1e-20**
29.2.1	protein.synthesis.ribosomal protein	446	< 1e-20
29.4	protein.postranslational modification	736	1.42E-19
29.1	protein.aa activation	94	3,15E-10
29.2.3	protein.synthesis.initiation	91	9,87E-10
29.2.4	protein.synthesis.elongation	34	2,42E-05
27.2	RNA.transcription	101	4,54E-04
29.2.2	protein.synthesis.misc ribosomal protein	14	2,81E-02
29.5	protein.degradation	2131	2,95E-02

Supplemental Table S2b. Wilcoxon Rank Sum Test followed Benjamini Hochberg correction among changes in transcript expression for MapMan Bins related to RNA-ProteinSynthesis in WT phase III over II (P< 0.05).

bin	name	elements	p-value
27.1	RNA.processing	379	7.86E-8
29.5	protein.degradation	2131	3.63E-4

Supplemental Table S3: Protocol for combined conventional and microwave-proceeded fixation, dehydration and resin embedding of *Arabidopsis* apical shoot meristem for histological and ultrastructural analysis.

Micowave processing in a PELCO Bio Wave®34700-230 (Ted Pella, Inc., Redding CA, USA)				
**Process**	**Reagent**	**Power [W]**	**Time [sec]**	**Vacuum [mm Hg]**
		150	60	0
	2.0% (v/v) glutaraldehyde and	0	60	0
**1. Primary fixation**	2.0% (v/v) paraformaldehyde	150	60	0
	in 0.05 M cacodylate buffer (pH 7.3)	0	60	0
		+ 12 h on shaker at RT		
**2. Wash**	1x 0.05 M cacodylate buffer (pH 7.3) and 2x aqua dest.	150	45	0
		0	60	10
		80	120	10
**3. Secondary fixation**	1% (v/v) osmiumtetroxide in aqua dest.	0	60	10
		80	120	10
		+ 15 min. on shaker at RT		
**4. Wash**	3x aqua dest.	150	45	0
**5. Dehydration**	Acetone series: 20 %, 30%, 40%, 50%, 60%, 70%, 80%, 90%, 2x 100%	150	45	0
	and 1 x propylen oxide	+5 minutes each on shaker at RT		
**6. Resin infiltration**	Spurr´s resin in propylen oxide 25%	2 h on shaker at RT		
	50%	2 h on shaker at RT		
	75%	2 h on shaker at RT		
	100% Spurr	12 h on shaker at RT		
**7. Polymerisation**	24 hrs at 70°C in Beem capsules in a heating cabinet.			
